# Ninth International Medical Congress

**Published:** 1887-10

**Authors:** 


### SECTION ON MILITARY AND NAVAL SURGERY AND MEDICINE.

FOURTH DAY—MORNING SESSION.

Dr. J. D. Bryant, of New York, N. Y., being absent, his paper entitled

ON THE CAUSES AND TREATMENT OF ERYSIPELAS, WAS READ BY TITLE.

A paper by Dr. William Varian, of Titusville, Pennsylvania, on the subject of

ETIOLOGY AND TREATMENT OF HOSPITAL GANGRENE DURING THE WAR, then followed. The author enumerated the symptoms of this disease as observed by himself during the late war, and the causes which were probably concerned in its development. There may be certain atmospheric conditions, such as the prevalence of cold, wet weather, or hot moist weather, with marked electric or barometric oscillations. The influence of bad food, imperfect care, and mental depression acted, the speaker said, as predisposing cause. The probability of the presence in the atmosphere of some unknown septic poison was discussed at length. A necessary condition for the development of the disease is the existence of a solution of continuity of the cuticle or soft tissues generally; the same may be said of wounds in a state of suppuration and granulation; wounds not recent being, however, more likely to be attacked. Hospital gangrene is a local, and not a systemic, disease; it develops an auto-generative contagium, which may be spread by atmospheric diffusion, or may be conveyed from patient to patient by the surgeon's hands, instruments or dressings. That it is a local disease is attested by the fact that it may develop in only one wound upon an individual afflicted with a number of them; a few such cases were observed during the late war by others as well as by myself.

The subject of treatment was then entered upon. The futility of medicinal treatment was shown, and the complete destruction of the tissues affected was, the author said, essential to successful treatment. These should be boldly removed by the knife, and some caustic thoroughly applied. Success is less dependent upon the nature of the latter than upon the thorough application. In his experience he had found bromine the surest, although the most disagreeable; but nitric acid and the acid nitrate of mercury had also been successfully used. The importance of a supporting treatment, of good food, stimulants, and tonics, to combat the great vital depressions which always co-exists was shown. In conclusion, the author remarked that strict asepsis should eliminate hospital gangrene from the surgery of the future.

Dr. Frederick Hyde, of Syracuse, New York, desired to know if the author had met with cases in which the disease had actually been communicated by unclean sponges and dressings; he had himself observed this in a number of instances. An affirmative answer was given.

Dr. Pickett, of Cory, Pennsylvania, related his personal experience with hospital gangrene; he had been treated by bromine and cured, but the disease had made such an impression upon him that he was still suffering.

The President, Professor Smith, asked one of the British members, Dr. Anderson, of the British army, what had been his experience with the disease. Dr. Anderson replied that he had never met with it in his hospital experience.

A paper on

THE ETIOLOGY AND TREATMENT OF CAMP DYSENTERY AND DIARRHŒA, was read by Dr. Charles W. Buvinger, of Pittsburgh, Pennsylvania. Camp dysentery and diarrhœa are always the result of a radical change in the manner of living; by the use of bad water from stagnant pools or wells contaminated with organic matters. The use of improperly cooked food, and privations, were important factors. The speaker adverted to the influence of malarial or paludal neighborhoods and to that of scurvy in their causation.

The nature of these diseases is not well understood. They are not due to a micro-organism, bacteria, or a special bacillus, so far as known; in spite of numerous researches, we are still in the dark as regards their origin.

The history of the treatment of dysentery shows the diverse and discordant opinions entertained by authors regarding the use of ipecacuanha. No one remedy can be considered a specific for either of the diseases in question. The writer having been a great sufferer from both, briefly related his experience and experiments upon himself with various remedies.

He is convinced that fuming nitrous acid, 43%, as furnished by Powers & Weightman, of Philadelphia, is the best remedy that we have; it may be reinforced by opium, if necessary. His favorite formula is the following:

℞. Acidi nitrosi, 43%.......... f ʒ j.

Tinct. opii.......... f ʒ ij.

Aquæ destil.......... f ℥ vi.

Syr. simpl.......... f ℥ viij.

M. Sig—A tablespoonful in one wineglassful of water every three hours.

This remedy will afford relief in the majority of cases of dysentery and camp diarrhœa. Oil of turpentine is most valuable in the latter disease, especially in the hæmorrhagic form as well as in the chronic diarrhœa met with in private practice. The writer has for years used with success the following formula, which possesses the advantage of being pleasant to take, and is readily taken by children:

℞. Acaciæ pulv.......... ℥ jss.

Ol. terebinth .......... f℥ ij. ʒij.

Aquæ.......... f℥ iij.

Syr. simpl.......... ad f℥ xij.

M. Sig.—One teaspoonful every three hours to adults.

It will be noticed that this emulsion contains twenty drops to the drachm, a quantity which is less likely to cause strangury than a smaller dose. It may be reinforced by tr. opii, to prevent undue catharsis. The dose for children should, of course, be proportionate to their age. He had found this combination invaluable.

In camp diarrhœa, another efficient combination is the following, which will be found useful also in summer diarrhœa:

℞. Hydrarg. chlor. mit.......... gr. ij.
Pulv. ipecac.......... gr. iij.
Opii.......... gr. v.

M. et in chartulas No. X. divide.
Sig.—One every three hours.

The next paper was read by Dr. Joseph R. Smith, U. S. Army, upon

THE BEST FORM OF REPORT OF SICK AND WOUNDED OF ARMIES.

The object of such a report was described, and the form adopted by various European armies shown. Of these, that of the Prussian army is, in the opinion of the writer, the best. A model of a form suitable for all armies, designed by the author, was presented.

Five propositions were formulated for adoption by the section, the first stating the objects of report of medicine and surgery; the second the frequency and periodicity of the same; the third recommending simplicity and uniformity; the fourth expressing preference for the Prussian system of classification; the fifth urging the adoption of the model form here presented, and directs the Secretary of the section to communicate their opinion to the medical bureau of all nations represented in the congress.

An essay by Dr. Chas. W. Brown, of Elmira, New York, upon

THE ETIOLOGY AND TREATMENT OF TETANUS

was read. The author began by saying that the cause of the disease is still enshrouded in doubt, but was more probably due to ascending neuritis than to the irritation of peripheral nerves. He considers the disease a specific, contagious, and infectious one, which, according to Rosenbach, is caused by a bacillus, his experiments having shown that rabbits and mice inoculated with material from a case of tetanus died of the disease. The author adverted to some experiments made by various observers upon the horse, and proceeded to compare a true case of the disease with one of cerebro-spinal meningitis, which, he thought, had often been mistaken for idiopathic tetanus; in fact, his experience and observation had cast a doubt in his mind as to the existence of such a disease as idiopathic tetanus, which was always the result of some wound, however slight. After an interesting account of a case met with in his practice he approached the subject of treatment, which he characterized, as a rule, as very unsatisfactory. The wound should be, if necessary, freely opened and cleansed with some antiseptic fluid, and dressed in accordance with the methods of modern asepsis. Isolation and rest in a dark room, free from air-currents, were of great importance. The removal of wounded parts has not given good results in traumatic tetanus. Supporting measures are indicated in the disease, and the intelligent use of stimulants. The use of concentrated foods was not to be overlooked.

Quinine had been in some cases, apparently, used to advantage, in enormous doses, some giving as much as two hundred and sixty grains in one dose. He had himself administered it in doses of one hundred grains every hour for some time; this case recovered, and no bad results followed from the injection of such a large quantity of the drug. Fowler's solution, cannabis indica, morphia, cocaine, the bromides, had in turn been tried in vain, although the latter, particularly bromide of potassium had been strongly recommended by Dr. McCord, of Kentucky, in twenty grain doses, every hour. The use of pounded ice and the ether spray along the spine should always be resorted to.

The President, Professor Smith, announced that the paper which had been promised upon

THE MICROBIC ORIGIN OF TETANUS, by William Browning, M.D., of Brooklyn, New York, had not been received. He then called upon Dr. J. McF. Gaston, of Atlanta, Georgia, to open the discussion upon this interesting subject.

Dr. Gaston, at the outset, regretted exceedingly that the paper just mentioned had not been presented, as he had expected from it, and as a result of the author's experiments, the presentation of new data of a definite nature upon this subject. In the absence of any such data, and relying upon his own experience with the disease, the speaker's views were not in harmony with those of Dr. Browning, concerning its bacterial origin, contagious, or infectious nature. Professor N. Senn, of Milwaukee, in a recent lecture, appeared to take for granted the bacterial origin of tetanus, the author of the paper under discussion appeared to hold the same views, but has not eliminated the doubt which may exist in regard to this etiological factor. It is begging the question to claim that tetanus is due to bacilla, because a certain bacilius of a distinctive character has been found in the course of the disease, since such an organism may be a mere accompaniment or result, and have no causative influence whatever. Regarding the claim of contagiousness or infectiousness, chiefly based upon the synchronous or subsequent appearance of the disease in man or animal in the same locality, it should be considered that there may be certain predisposing atmospheric conditions in such locality which account for the occurrence of the disease without implying its infectious or contagious nature in the ordinary acceptation of the terms.

In the speaker's experience, suppurating wounds do not often cause tetanus. The disease may be due to the development of some ptomaine, but the question of its origin is still *subjudice.*

As for the treatment, the use of chloroform, and the thorough saturation of the system with chloral and potassium bromide offer the best chance of success. The speaker had observed eighteen cases of traumatic tetanus, treated in this manner, of which ten cases had recovered.

---

AFTERNOON SESSION.

The first paper of the session was read by Dr. J. W. S. Gouley, of New York, N. Y., on

THE PRACTICAL CONSIDERATION OF HUMAN NOSOGRAPHY.

The author first considered the importance of a correct and thorough nomenclature of diseases, and the causes that had retarded the progress of this science. To be of practical utility, nosography should be established upon a sound basis. The defects in the past and present nomenclature of diseases were chiefly enumerated, and suggestions given for their remedy. The paper concluded with the presentation of propositions looking toward an improved classification of diseases.

Dr. Simeon Tucker Clarke, of Lockport, New York, spoke of the importance of this subject, and agreed, in the main, with the views of the author.

Dr. Harrison, of Washington, D. C., Dr. Didama, and Dr. Frederick Hyde, of Syracuse, New York, discussed the subject and presented their views relative to its importance.

Dr. Hingston, of Montreal, Canada, spoke of the necessity of such a revision as that proposed, and Dr. Bontecou, of Troy, New York, referred to the simplicity of the Prussian nomenclature of diseases, as compared with ours.

Dr. Gouley concluded the discussion by suggesting that co-operative movement was needed to reach the desired end of a perfect nomenclature.

The second paper, entitled,

WHAT CLASS OF GUN-SHOT WOUNDS AND INJURIES JUSTIFY RESECTION OR EXCISION IN MODERN WARFARE?

was read by the author, Dr. Reed Brockway Bontecou, of Troy, New York.

After a clear definition of the terms, the author entered upon a consideration of the value of operations as compared with the results of expectant treatment or amputation, referred to the advantages of excisions and their reduced mortality under the antiseptic method, and gave statistics of gun-shot wounds of cranium, clavicle, scapula, and shoulder-joint. The fractures of the hip-joint were next considered. The results of resections of lower extremities were not satisfactory with regard to their future usefulness. The condition demanding excision of the shaft of femur in gun-shot fractures were chiefly enumerated. In conclusion the author adverted to the favorable statistics of antiseptic treatment without operation, and to the encouraging results of resection of the knee-joint under the same method of treatment.

Professor H. E. Goodman, of Philadelphia, after few remarks upon the paper, reported some cases of resection of the femur after the battle of Chancellorsville, attended with fatal result.

Dr. Henry Janes, of Waterbury, Vermont, read an important paper, giving the results of his experience during the late war, with

GUN-SHOT FRACTURES OF THE FEMUR.

The author's conclusions upon this interesting subject are based upon the results of no less than 427 cases occurring in his service, and treated by himself, or under his immediate observation, in two field hospitals in his charge during the years 1862 and 1863.

Of these fractures 95 were in the upper third of the bone, all of which were treated conservatively, giving a mortality of forty-six per cent. at last reports; 125 were in the middle third, 102 of these being treated conservatively, with a mortality of thirty-two per cent.; 23 were amputated, mortality forty per cent.; 207 cases were in the lower third of the bone; 67 of these, not including knee-wounds, were treated by the conservative method, with a mortality of twenty per cent.; 140 cases were amputated, resulting in one of thirty-five per cent. Besides these 427 cases, there were 34 fractures involving the knee-joint, treated conservatively, with a mortality of eighty-five per cent. The cases requiring amputation terminated earliest, but about one-eighth of them suffered from painful and tedious osteitis. Up to about forty years of age, advance in life did not seem to increase the rate of mortality. The author's conclusions are the following:

Antero-posterior wounds did no better, in my experience, than the transverse. Fractures caused by bullets moving with great velocity were fraught with more danger than those at a lower rate of speed.

Wounds occurring in prisoners did not do so well as those in their captors under the same conditions; this was probably due to the incident moral influences.

Gun-shot fractures sometimes united as readily as simple fractures.

False joint occurred but once among the two hundred and sixty-three cases treated conservatively.

Secondary hæmorrhage occurred only in nine cases, several times incident to septic poisoning.

Tetanus only occurred once.

Hospital gangrene seemed to be induced in the autumn of 1863 by bad ventilation and the occurrence of cold, damp weather.

The shortening, in the cases which completely recovered was generally of more than one inch; it was, in several instances, increased by too early use of the limb.

The formation of callus was sometimes so irregular and excessive in amount as to impede muscular action; it sometimes encroached upon an artery or nerve, and caused secondary hæmorrhage or neuralgia. In some cases the callus was absorbed in the course of septic inflammation.

Refractures, caused by falls and similar accidents, generally reunited readily when the wounds in the soft parts were healed.

The limbs often became much more useful than any artificial substitute; the author has unfortunately no statistics bearing upon this subject to offer.

Regarding the treatment of injuries of this nature, he would enjoin the surgeon *not* to saw off the ends of fractured bones, and not to use too much force in tearing off fragments of bone still attached to the periosteum. Moderate, simple extension by weights and pulleys is the best in the majority of cases; in some of the cases that did the best no extension was used. He would advise the dry dressing of wounds, with plenty of absorbents rather than with water-dressings. Strict antisepsis, according to the modern idea, is impossible in large hospitals on the battle-field.

In conclusion, the author spoke of the necessity of a liberal diet, the intelligent use of stimulants, and due regard to the sanitary precautions in the camp or hospital in which this class of injuries are treated.

Dr. Robert Reyburn, of Washington, D. C., desired to know what proportion of Dr. Janes' cases had been treated in tents and in buildings.

Dr. Janes replied that the majority of cases were treated under canvas, with free access of air; with the advent of the cold and damp weather of winter the ventilation was not so perfect, as it was difficult to impress upon the soldiers the importance of free ventilation at a time when they were suffering from the cold. It was, as previously stated, at this time that hospital gangrene set in.

Dr. Reyburn said—In my experience the kind of hospital in which the wounds under consideration are treated has much to do with the mortality; the treatment in tents I consider the best; unfortunately, most of the cases observed by him in the service could not be treated under such favorable conditions, and he had to report a mortality of about seventy-five per cent. The results achieved by Dr. Janes were most remarkable, and show what can be done by conservative surgery.

---

### SECTION ON OBSTERICS.

SEPTEMBER 8TH — FOURTH DAY — MORNING SESSION.

Professor Alexander R. Simpson, of Edinburgh, Scotland, occupied the chair.

Dr. Emil Poussie, of Paris, France, made a report of a case of

### TYPHOID FEVER IN THE PUERPERAL WOMAN

Seven days after labor, grave symptoms of true typhoid fever appeared; the disease running its course and the patient recovering. The report was made to call attention to the importance of distinguishing typhoid in the puerpera, from septicæmia.

Professor Alexander R. Simpson called attention to the importance of distinguishing the various forms of puerperal fevers. In Edinburgh he saw many cases of typhoid after labor; these were sometimes difficult to differentiate from ordinary sepsis, sometimes distinct and with a clear history of sewage-infection. He had seen scarlatina and measles after delivery simulating septicæmia. These diseases in the puerperal woman were apt to prove fatal before their full development.

Professor Graily Hewitt, London, England, thought that the great advance in the treatment of puerperal fever in late years consisted in means tending to prevent the introduction of septic material into the blood. Women in whom the uterus did not remain firmly contracted were the most liable to contract puerperal fever. The uterus was the seat of entrance of the poison, and in private practice the best safeguard was to keep the uterus firmly contracted. Keep the patient in as good general health as possible during the latter months of pregnancy, feed her soon after labor and keep up her strength, for where there were weakness and debility the uterine contractions were apt to be imperfect. He used antiseptic precautions and vaginal injections, with the uterine douche in appropriate cases. When the germs had once gotten into the connective tissue about the uterus, douching did no good. He insisted upon the necessity of free passage for the return flow, and advocated Budin's double-tube, which he had had made of glass, as it was then more easily examined and kept clean. In general treatment keep up the uterine contractions by various means, nourish the patient, and use stimulants *freely*.

Dr. G. W. Jones, of Danville, Illinois, had never had a case of puerperal fever in his own practice. He is always careful regarding antisepsis, employing the mercuric bichloride, or oil of turpentine, which he considered an excellent antiseptic properly used. The uterine douche is a valuable agent.

Dr. William T. Stewart, of Philadelphia, Pennsylvania, read a paper on

### THE IMPORTANCE OF ACCURATE DIAGNOSIS IN PREGNANCY, WITH THE HISTORY OF A UNIQUE CASE OF RETROFLEXION OF THE GRAVID UTERUS, LABOR AT TERM.

After some remarks on the causes and necessity of the numerous cases of abdominal surgery, in which he expressed a hope for some better means of prevention of female diseases, he spoke of the evil effects of the modern steel-clad corset, which injured the muscles of the back, forced down the contents of the abdomen, and impeded the venous return. He reported a case showing many blunders in the way of mistaken diagnosis.

The patient, aged twenty-nine, one year after the birth of her third and last child consulted a female doctor, who told her that a tumor was growing in the posterior vaginal cul-de-sac. She consulted several other physicians, some of them famous gynecologists, who agreed as to the nature of the "fibroid" and decided upon operation. Two days before the proposed removal of the tumor Dr. Stewart chanced to see her, and found a retroverted pregnant uterus. He advised her not to undergo the operation, and, as she was not his patient, did nothing more. At full term he was called upon to deliver her, and found the uterus still retroverted. The vertex presented above the brim, the body lying posteriorly. The os was dilated, but nearly out of reach above the pubes. An attempt at reposition, made with the patient in the knee-chest position, was successful. At the first pain after the reposition the membranes were ruptured, and at the second the child was born, living; weight, six and a half pounds.

Only two similar cases were recorded, in both of which the child was dead.

Professor Alexander R. Simpson, of Edinburgh, Scotland, thought the case interesting, as it was extremely rare for a retroversion to persist to term.

Dr. Rodney Glisan, of Portland, Oregon, related the history of a case of retroversion of the gravid uterus, reduced in the knee-chest position at the fourth month, and followed by abortion. We should always insist upon a rectification, even to the fifth month.

Dr. Williams, of Baltimore, Maryland, related a case where a retroverted gravid uterus, with bilateral laceration of the cervix to the vaginal junction, simulated cancer with fibroid.

Professor John Bartlett, of Chicago, Illinois, presented a paper entitled

A STUDY OF DEVENTER'S METHOD OF DELIVERY OF THE AFTER-COMING HEAD, supplementing the paper with a demonstration upon the phantom.

Deventer spoke in the most confident manner of the success and safety of podalic version, and of the ease with which the head could be delivered; but did not describe his method, which, however, Dr. Bartlett had found mentioned in Smellie's work. Deventer's method was shown to consist of a reversal of the so-called Prague method, in that the body of the child was carried far backward toward the perineum, with the view of turning the occiput out from under the pubes, the anterior surface of the neck resting on the perineum. At the beginning the occiput of the child was turned forward, so as to come under the pubes as the child was drawn down. The arms were *not* to be drawn down, but left up alongside the head, being placed so as to come anterior to either parietal base. The delivery by traction backward upon the body was to be aided by pressure made immediately above the pubes, the wedge formed by the head and arms being changed by the withdrawal of the larger transverse diameter of the head from between the arms, as descent of the head accompanied by extension occurs. The mechanism was only favorable when the occiput was anterior. Deventer never lost a child or tore a mother. The arms being left up protected the neck of the child and allowed a passage for the cord alongside of them, so that haste was not as necessary as with ordinary methods, and, occupying a broad and yielding part of the pelvis, they did not obstruct delivery. The method was a plausible one, and certainly worthy of trial in suitable cases.

Professor Simpson spoke of Deventer as one of the most reliable of obstetric writers; yet, as the method would seem to endanger the perineum, he would like to see a practical demonstration of its value.

Professor A. F. A. King, of Washington, D. C., considered Professor Bartlett's paper one of the best that had been presented to the section. In regard to the safety of the method, we must remember that the puerperal canal was elongated by the perineum, which he thought liable to be torn. However, he should describe the method in the next edition of his book.

Professor C. T. Parkes, of Chicago, Illinois, described two cases where he had had difficulty in getting down the arms, and where a decided pull backward had easily and unexpectedly delivered the head. He had not understood the matter until Professor Bartlett's paper had made it clear.

Dr. G. W. Jones, of Danville, Illinois, cited three similar cases where he had, without knowing it, used this method, with the patient on the side, the delivery being unexpectedly easy and the perineum intact in each case.

Dr. J. E. Kelly, of New York, read a paper entitled

LITHIASIS IN PREGNANCY.

Being impressed by the frequency, during gestation, of many of the arthritic, gastric, and other phenomena which ordinarily are present in lithiasis, the author reviews in detail the relations existing between the two conditions, and concludes that the association is due to the correspondence of the condition of the blood in lithiasis and pregnancy. First, considering the general pathology of lithiasis, and subsequently that of the various systems, he then investigates the phenomena of pregnancy, and indicates the influences which produce in the maternal blood a condition almost identical with that which is present in lithiasis, and draws a parallel between the diseased conditions most frequently observed in pregnancy and the symptoms of lithiasis. These investigations would suggest the careful supervision of the condition of the pregnant female, especially with regard to the digestive, circulatory and urinary symptoms, and, in suitable cases, the intelligent application of prophylaxis.

Dr. E. P. Christian, of Wyandotte, Michigan, read a statistical paper on

THE PROPORTION AND CAUSES OF STILL-BIRTHS.

The average for states and counties was about four per centum; for large cities, seven per centum. His personal statistics, in a small manufacturing town, were a little less than four and a half per centum in 1,675 labors, including 17 cases of twins.

Prominent among the causes of mortality were syphilis, intemperance and ergot.

Professor Alexander R. Simpson spoke of the value of such statistical researches, and of the labor they required. He strongly condemned ergot, given before the birth of the child, as being a most fruitful cause of still-births.

Drs. Dunmire, of Philadelphia, Pennsylvania; Pierce, of Ohio; Lester, of New York, N. Y.; Stewart, of Philadelphia, Pennsylvania; Robinson, of Danville, Virginia; and Sale, of Mississippi, united in condemning the use of ergot.

### SECTION ON GYNÆCOLOGY.

SEPTEMBER 8TH — FOURTH DAY — MORNING SESSION.

Dr. Ephraim Cutter, of New York, N. Y., read a paper on

GALVINISM OF UTERINE FIBROIDS.

The paper was divided into Expectations, Realizations, Answers to Critics, Conclusions.

EXPECTATIONS.

August 21, 1886, the first American operation was performed, simply to arrest development of the fibroid. It was participated in by Dr. W. S. Stoveham, of Massachusetts (*American Journal of Obstetrics*, New York, February, 1887, 120).

Dr. Gilman Kimball was present, August 29, 1887, and operated the second time. "So far as the priority is concerned, I am willing to give it to him, for I will have no question of honor between him and me."

The idea of using common needles was scouted at once by their unsatisfactory performance. Dr. Kimball said he would have nothing to do with the operation unless better needles were provided. I invented one plated with gold and shaped like a corkscrew, which proved a useless device. The so-called Cutter needles were then produced. Whatever has been or may be said of them, they were satisfactory.

REALIZATIONS.

After sixteen years, besides the expected arrested development in a large majority of the cases, there have been: 1, entire cures; 2, great diminution of the growths; 3, relief from pain and hæmorrhages; 4, attention to the operation by the profession; 5, about four hundred cases of operation reported, besides many scores not reported; 6, the variation from galvanism to faradism, in the mode of application, in the batteries, in the duration of the application, in the kinds of electrodes, in the discovery of instruments for mensuration; 7, the operation has been widely published and has become pretty well known; 8, uterine fibroids are no longer opprobia medicina.

When the first operation was done there were no amperemeters; only voltameters, which are measures of electro-motive force. It is not claimed that the milliamperemeter cures; it only measures. I only object to the doctrine that no operation can be done without measurement, not to measurement itself.

APOSTOLI'S CRITICISMS.

I distinguish between him and his sayings; I know him for the work he has done, and reply to him in no spirit of malice.

CONCLUSIONS.

1. When an untried operation is purposed, it is not wise for those whose opinion is asked as experts to say it cannot be done.

2. Best to give all due credit for what they do.

3. In performing this operation use common sense, and not blindly follow any one's method unless indicated by the strongest signs.

4. Hereafter any physician who says uterine fibroids are hopelessly incurable is not sustained by the facts and evidence.

Dr. A. Lapthorn Smith, of Montreal, Canada, was sorry that Dr. Apostoli could not defend himself against the criticisms in the paper of Dr. Cutter in the English language. Most uterine fibroids are situated posteriorly, and hence are easily reached and treated by negative galvano-puncture. I have been deluged with questions about this treatment since the reading of Dr. Apostoli's paper and my own. As to the question, Can fibroids be treated without puncture? I would refer you to Dr. Martin's paper, which was on this subject. Apostoli never dares to make an examination without first washing out the vagina with a bichloride solution (1 in 1,000, 2,000, or 3,000). He cures subinvolution, and with the positive galvano-cautery does away with dilatation in very many cases of dysmenorrhœa. Gentlemen who make their living by the removal of the ovaries will lose their occupations when electricity is more in vogue.

Dr. F. H. Martin, of Chicago Illinois, said he knew it to be impossible for Dr. Cutter to get any current at all through the battery described by him.

Dr. Kimball, of Lowell, Massachusetts, who was introduced by the president as a pioneer in the treatment of fibroid tumors by electricity, gave his experience.

Dr. Garrett, of Boston, Massachusetts, who was also introduced as a pioneer, gave his testimony regarding this treatment.

Dr. Apostoli, of Paris France, spoke in defense of his system. He wished to give Dr. Cutter all credit for his investigations; but now that we have the means of measuring electricity, a measurement made definite by the electrical congress of Paris, there is no excuse for using it hap-hazard and every one should use it rationally.

Dr. Smith, of Montreal, Canada, then said there were a hundred little details, which it is impossible to explain in the short time allowed.

Dr. Cutter asked Apostoli if he had cured any case so that the tumor had entirely disappeared.

Dr. Apostoli replied that he cures the cases symptomatically, the patient does not know any more that she has a tumor.

---

### Afternoon Session.

Dr. August Martin, of Berlin, Germany, read a paper on

### THE VAGINAL TOTAL EXTIRPATION OF THE UTERUS FOR CANCER.

Freund inaugurated the extirpation of the cancerous uterus ten years ago. Sufficient material is now at hand to decide the two following questions, which may legitimately be asked concerning every new method of surgical treatment:

1. Is this operation practicable with such immediate success that it promises good results in the hands of others than a few specially successful operators?

2. Does the extirpation of the cancerous uterus give permanent results which force us to recognize that this method is superior to any other treatment of cancer employed up to the present time?

In seeking an answer to the first, if we examine the literature, we are struck with the fact that only meagre and isolated reports about this operation can be found in English and German medical journals. Vaginal extirpation has obtained decided recognition in Germany. In this country the purely vaginal operation of Czerny and Billroth and Schroeder has been adopted instead of the procedure of Freund, which was a combination of abdominal and vaginal operations. The results of the same have improved noticeably with increasing exercise and experience.

In 1881 Olshausen collected 41 cases with twenty-nine per cent. mortality. In 1883 Sanger, 133 cases, twenty-eight per cent. mortality. In 1884 Engstrom, 157 cases, twenty-nine per cent. mortality. In 1886 Hegar, 257 cases, twenty-eight per cent. mortality.

Through the courtesy of these operators, who to my knowledge commanded the greatest amount of material, and, at my request, placed at my disposal the results up to the end of the year 1886, I am able to present the following:

Up to the end of 1886 these total extirpations have been performed on account of carcinoma uteri: Fritsch, 60 times with 7 deaths; Leopold, 42 times, 4 deaths; Olshausen, 47 times, 12 deaths; Schroeder (Hofmeir), 74 times, 12 deaths; Staude, 22 times, 1 death; A. Martin, 66 times, 11 deaths. Total, 311 cases with 47 deaths, or 15.1 per cent.

Are we not justified in assuming that this

RATE OF MORTALITY WILL DECREASE, with more experience, as shown by the published tabular results of each of these operators? Already the total extirpation of the uterus for cancer shows better results, so far as immediate mortality is concerned, than removal of the breast for cancer.

For the latter, Kuster, at the twelfth meeting of the German Surgical Society, in 1883, published 778 cases with a mortality of 15.6 per cent. Who would hesitate to propose to perform the amputation of the cancerous breast so soon as the diagnosis is established?

I do not hesitate to answer my first question in the affirmative, and to claim for this operation of the vaginal total extirpation of the uterus a full and equal rank among all methods for the treatment of cancer of this organ.

For an answer to the second we will make use of the relatively small, but very accurately reported cases of Schroeder, collected by Hofmeir, and those of Fritsch, Leopold and myself.

Table II. shows that the permanent results of vaginal total extirpation in this relatively short period of observation are no doubt equal to the best results of carcinoma operations of other organs.

The author up to the end of 1885 operated on 44 cases. Of these relapsed 18, or 29.7 per cent.; recovered, 31, or 70.3 per cent.

Is there any other method of treating cancer which with so small mortality can show equally good results? There is no other mode of treating cancer of the fundus and those forms of diseases of the cervix in which the mucous lining of the cervical canal is the point of origin, or in which there are carcinomatous nodules in the tissues of the neck. There is no room for discussion except in epithelioma of the portio vaginalis arising from the surface of the cervix, that is, from a surface covered with flat epithelium and containing very few glands.

I agree with Fritsch that the observation of cases of progress of the disease in isolated nodules in the mucous membrane up to the fundus, in cases of carcinoma colli, is sufficient in itself to show it is erroneous to claim that in carcinoma of the cervix we should try to save the body of the uterus. Binswanger and P. Ruge have described such well-marked cases.

The possibility of a subsequent pregnancy is not excluded in cases of high excision; but Hofmeir himself declares that pregnancy is a very serious danger in carcinoma. Therefore, I am convinced that it is much better to immediately perform vaginal total extirpation in these forms of epithelioma of the cervix. The sooner we operate the more surely we may hope to save our patients from the sad fate of death by cancer. The greater the experience with vaginal total extirpations the more has the rule been proved that we shall perform the operation only when the vicinity of the uterus is entirely free from carcinomatous infiltration. All attempts to enlarge the boundaries of the operation in this direction have failed.

The technique of the operation has undergone only immaterial changes, as is shown by the results of operators using different methods. It is irrelevant whether the uterus be removed by an incision made in front of, or at the side of the neck, or behind it. It is of little importance whether hæmorrhages be prevented by stitches introduced before the incision, or whether each separate vessel be seized and tied as it bleeds. It is immaterial whether the uterus be turned over or removed by drawing it down and freeing it, whether the opening in the floor of the pelvis remain open or be closed, or be drained either with the iodoform gauze or with a tube.

If it be easily practicable I advise that the ovaries and tubes be also removed. It is immaterial whether the wound be sutured or not. It is wonderful what little impression the operation makes on the patient. One can liken her very mnch to a puerperal woman.

Bleeding must be stopped, at all events during convalescence; the parts as much as possible kept at rest. Washing out the peritoneal cavity does not work favorably.

The papers of Drs. Martin and Jackson on "Hysterectomy for Uterine Cancer," were discussed.

Dr. Martin, of Berlin, Prussia, said: I am accustomed to prepare my patients for operation with the most thorough antiseptic vaginal injection. He then described most minutely his method of operating. He very frequently opens the Douglas pouch at one stroke of the knife. You then see the posterior fornix and cut carefully, paring with the finger-nail. When the peritoneum is opened I introduce one finger into the peritoneum and, having warm water running over the surface, do not use sponges. I suture the vagina to the peritoneum. When I have freed the broad ligament I cut it from the uterus and generally have no hæmorrhage. I then proceed in like manner on the other side, till I have the broad ligament severed there also. Up to this stage there is very immaterial hæmorrhage. I then commence on the bladder—freeing it with the forceps or the knife. After it is freed I unite the cut border of the vagina with the peritoneum, just as I did before. I am accustomed to put a drainage-tube into the peritoneum, consisting of india-rubber. I think it does good, although I must confess that I believe that a case which is not infected, should be closed up. Yet I have had such good results from this that I still use it. There are various gentlemen present who have seen me operate, and who can testify that I lose a very small amount of blood. Of course there are cases where a loss of blood is necessary from the existing conditions. The operation is yet new, it has only been done for six years, and I think we will improve on it very much.

Dr. D'Arnay, of Hungary, gave a history of his experience with twelve cases. Out of these twelve cases only two are now living, after a period of three or four years. As to the operation he thought that the best we could do was to close the opening into the pelvis, and fill the vagina with iodoform gauze, and let the patient alone. As to Dr. Jackson, he thought he was on the wrong road with his statistics. Statistics deal only with quantity, not with quality. If there are one hundred persons on a ship, possibly I can save only one of them. Should I not save this one? Certainly. Patients suffering with cancer of the uterus are shipwrecked persons, and sure to die a most painful death. We can save a number of our patients, and those who die usually die in a short time, and comparatively comfortable deaths. Humanity demands of us that we do the operation of vaginal hysterectomy in all cases in which we can remove all of the diseased tissue.

Dr. A. P. Dudley, of New York, N. Y., wished to enter a plea for vaginal hysterectomy for uterine cancer in America, and point out why the operation had been less successful here than in Germany. Martin had sixty-six cases, with eleven deaths. In a paper read by me, some time since, I reported sixty-six cases with thirty-four deaths. These sixty-six cases, however, were divided between thirty-four operators, and here lies the difficulty. Experience is a good teacher, and practice makes perfect. The child must creep before it can walk, and the surgeon must have a fair trial, especially in America. As in the case of ovariotomy, which originated in America, our surgeons are almost flocking to Europe to learn how it is done. The amount of pain and suffering in the death of the patient is one of the points which should induce us to try this operation.

Dr. Nunn, of Savannah, Georgia, said that there was always a starting point to cancer. It is generally due to neglect of fissures or some other irritant, arising consequent to parturition In my own practice I have my patients report to me occasionally after delivery, and see that they take care of themselves properly, and I have not had a case of cancer in my own patients.

Professor Graily Hewitt, of London, England, said that the whole civilized world, and the uncivilized too, are under obligations to Dr. Martin and his colleagues for their work in this line, having advanced the operation to its present state. In a discussion in the London Obstetrical. Society, a few years ago, I was the only one who refrained from condemning the operation. I think it should be done in properly selected cases, by gentlemen of experience.

Professor A. Reeves Jackson, of Chicago, Illinois, thought it wrong to attempt to reason against facts, as well as difficult. His paper was based on facts founded on the results of Gusserow, Paget and such men. These gentlemen had estimated the duration of life, in those women suffering from uterine cancer, to be twenty-one months as an average. If this be true, then my calclation as to the duration of life will not be denied. The average duration of life of women operated upon is fourteen months; this difference in the aggregate amounts to nearly three hundred years of grand total loss of life. Does anybody allege that it has saved life? Dr. Martin claims that the operation should be done only when the disease is limited to the uterus. How does he know the case was limited to the uterus? Because it did not return? Baker, of this country, by his high operation, has sixty per centum of recoveries, which is far better than Martin's.

Dr. Martin replied that he knew that a cancer was limited to an organ by its having a layer of entirely healthy tissue around it.

---

### SECTION ON THERAPEUTICS AND MATERIA MEDICA.

### Fourth Day.

A paper on

THE CHEMISTRY AND PHARMACOLOGY OF THE NITRITES AND OF NITRO-GLYCERINE, by George Armstrong Atkinson, M. B. C. M.' was presented, and in the absence of the author, was read by Dr. Ralph Stockman, of Edinburg, Scotland.

The action of the salts of nitrous acid resembles closely those of the acid which is the essential basis of this group of medicinal agents. Nitrous acid is remarkably unstable; in watery solution of $\frac{1}{8000}$ it may be used for a day or two for experimentation, but it has no advantage over a solution of nitrite of sodium, which possesses identical effects in so far as an acid can be considered identical with one of its salts. Our knowledge of the action of the nitrite group has been chiefly derived from a study of the effects produced by nitrite of amyl. Since here the base (amyl) has a decided action of its own, it is necessary to select a salt in which the base in its combination possesses no well-marked physiological activity. The resemblance between the action of sodium nitrite and amyl nitrite has been pointed out by Gamgee, Lauder-Brunton, Hay, Leech, and others. Barth described its highly poisonous qualities. Binz showed that it caused death from general paralysis, especially of the muscular system, no convulsions preceeding the fatal issue. Reichert considered it identical in its toxic effects with potassium nitrite. Its effects may be summed up as a paralyzer of muscular tissue, non-striated muscle being affected less quickly than striated. The brain centers are also affected. The blood becomes of a chocolate color in mammals (methæmoglobin), respirations are slowed, temperature slightly lowered. Death occurs in frogs from cessation of respiration; after the heart has stopped its movements, it is found in full diastole and quite inexcitable.

Post-mortem rigidity comes on early. In rabbits, three grains were a fatal dose in one three pounds in weight. The same appearances were found post-mortem. In man, small doses (eight grains) produced great tendency to faintness and considerable acceleration of pulse, and decided lowering of arterial tension. Paralysis of respiration is due to the effect of the nitrite on the muscular system chiefly, but also in part to the effect on the medullary centre. Small doses slightly increase the flow of urine; large always diminish it. Urea and uric acid are almost unaffected. Sugar appears in the urine of rabbits after some hours, if the animal be kept very decidedly under the influence of the drug; but rapidly disappears if the administration of the drug be stopped. The nitrite is largely destroyed in the system, being partly, however, excreted as nitrate, partly, probably, as urea; a portion of it is excreted as nitrite.

The pharmacology of the other nitrite is briefly dismissed. Nitrite of potassium, nitrite of ethyl, nitrite of amyl act in very similar manner to the sodium salt. Nitro-glycerine acts partly as a nitrite, and partly *per se*. In small doses it exerts the nitrite effect as a paralyzer: in large doses it produces convulsions.

Dr. Murrell, of London, England, referred to his discovery of the usefulness of nitrite of amyl in the condition of angina pectoris, and he always advised patients to carry the medicine in a small bottle. The pearls he considered too expensive for use. The tabellæ (Ch. B.) of nitroglycerine, made with chocolate, he considered dangerous from their resemblance to confections. He had used nitro-glycerine in one per cent. solution (dose m. v–xv) in cases of neuralgia of the fifth nerve, surgical shock, asthma (spasmodic and cardiac), and reflex neuroses. He preferred this in epilepsy to the nitrite of sodium, which had been recommended by Dr. Law. In angina, patients can take fifteen minims when they feel the attack coming on; such patients should carry a small bottle with them for immediate use when they feel the attack coming on, otherwise they might perish before the agent could be obtained. He gave an amusing instance of the difficulty of obtaining nitro-glycerine in England at present.

Dr. Upshur, of Richmond, Virginia, reported a case in which the inhalation of nitrite of amyl, in a patient suffering with heart failure and puerperal septicæmia, undoubtedly saved life by cautiously continuing the remedy, whenever there was a failure of the pulse, for about forty-eight hours, when it was withdrawn and diffusible stimulants substituted.

Dr. Wade, of Holly, Michigan, prefers a ten per centum alcoholic solution of the amyl nitrite, which he uses by inhalation. He regards it as the best form of cardiac stimulant in sudden emergencies.

Dr. Phillips, of London, England, had experienced headache, dizziness, and faintness after taking five minims of a one per centum solution of nitro-glycerine hypodermically. He had found the tabellæ (each m j. of a one per centum solution) useful in angina pectoris. Patients usually will not tolerate more than three or four of these, but some will require twenty-five or thirty. The effect of sodium nitrite is more prolonged than for the ethereal nitrites, and for this reason he prefers to give it in dyspnœa attending bronchitis.

Dr. Brackett, of Washington, D. C., reported a case of successful treatment of an epileptic by the use of amyl nitrite by inhalation.

Dr. Wade, of Holly, Michigan, recommended nitro-glycerine in cases of threatened local asphyxia of brain from embolism or thrombosis; he had had good results.

Dr. Phillips, in answer to a question, said that he would regard fifteen minims of solution of nitro-glycerine, hypodermically given, as a dangerous dose.

Dr. Murrell thought that there was no advantage to be derived from its hypodermic use, since it acts so quickly when swallowed.

The next paper was

ON THE POISON OF THE COBRA, by Dr. Julius Gnezda, of Berlin. The substance upon which the experiments were made was brought from India by Dr. Kobert Koch. It was obtained by making the cobras strike some shells covered by sheepskin. The poison was afterward dried. It is soluble in water, but not in alcohol or ether; its activity is lost by boiling. It is uniformly poisonous to higher animals. The European hedgehog and the ordinary swine of this country are popularly believed to enjoy an immunity, but this is explained by mechanical obstacles to absorption in these cases. The poison is contained in an albuminous fluid, but its chemical construction is not as yet settled. It is poisonous when applied to mucous surfaces, without causing blisters.

The effect is very marked when the poison is injected into the circulation. The blood-pressure is at first increased, the blood-corpuscles are changed in their shape, and the spectrum of the blood is characteristic. It was found that death did not occur at once, but after about half an hour and as the result of failure of respiration, so that the Indian Government was induced to recommend artificial respiration in such cases. At present, no antidote is generally accepted. Since it is not known whether the poison is an alkaloid, a ptomaine, or other body, such a question is at present only a speculation. As it is secreted by salivary glands, it is probably an albuminous body. Permanganate of potash has not been found an efficient antidote.

Dr. Phillips, of London, England, regarded the paper as an interesting contribution to our knowledge of the characteristics of snake-poison.

Dr. Lewin, of Berlin, Prussia, could not harmonize the report made of the spectroscopic appearance with the usual characters of the blood-spectrum. The appearance was peculiar, and needed further investigation. With regard to the antidote, it may be acknowledged that there can be no general antidote to counteract the poison after it gets into the system ; but if the remedy can be injected *locally* into the wounds, then potassium permanganate and a number of agents having a caustic effect may neutralize or destroy it.

Professor F. Woodbury, of Philadelphia, Pennsylvania, said that in regard to the nature of the poison, Drs. Mitchell and Reichart, of Philadelphia, had conducted a series of experiments which seemed to establish the fact that the snake-poison is not a single substance, but is probably composed of two, one of which is of the nature of a peptone. In the "Life of Francis Buckland," the English naturalist, the incident is given of Mr. Buckland having been inoculated with a very minute portion of the virus, accidentally, under his finger nail. He shortly afterward suffered with intense pain at the back of his head, faintness, and collapse, from which he recovered with difficulty after taking large doses of ammonia and brandy. He attributed his recovery to the antidotal effects of these agents. We may conclude that when a minute amount has been absorbed, ammonia and alcohol are antidotal, but where any considerable amount is received into the circulation the case is hopeless.

Professor Trail Green said that he had made some experiments with snake poison, and had been surprised by the quickness of the effects on a rat which had been bitten over the jugular vein. Death was instantaneous where the poison entered the blood directly.

Dr. L. Lewin, of Berlin, Prussia, read a paper

ON THE MAXIMAL DOSES OF DRUGS.

Of the many difficulties against which pharmacotherapy has to strive, the dosage of medicine is by no means the least. Variation in dose arises

(1) From the differences (*a*) between one person and another ; (*b*) between single individuals at different times ; (*c*) between the intensity of the disease in men suffering with the same affection ; (*d*) between diseases in which the same drug is used.

(2) From the variability in the activity of the greater part of our remedies.

At the same time it is possible and desirable to establish the usual dose of agents which are active in relatively small quantity, and which readily produce toxic effects when the dose is increased. Two groups of preparations fall under this head : (1) Plants (crude) and plant-products, and their pharmaceutical representatives ; and (2) chemical substances such as metalloids, metallic salts, and carbon-compounds.

The first group is almost universally inconstant in its effects, and thus far an international agreement as to their maximal doses has not been possible. The remedies of the second group, on the contrary, are nearly uniform, and the doses beyond which they cannot be given without danger can be determined by physiological experiment; but although the basis for such agreement is tolerably broad, yet the statements given in different pharmacopœias in many cases vary greatly. Such variations can be easily explained in the case of the first group, but becomes incomprehensible in the second.

From the results of his own observation and a comparison of the most of the pharmocopœias which give maximal doses, the author had constructed a table of the ordinary maximal doses of many of the drugs belonging to the second group, which he appended to this communication. Such a list should be added to the pharmacopœias of such countries as have have no maximal dosage, for the convenience of the practitioner, who thus would be enabled to prescribe such drugs with confidence even if they should not be generally used in his own country. From this Congress he hoped the influence would go out which would make such an international agreement possible.

Professor F. Woodbury, Philadelphia, Pennsylvania, instanced the varying effect from chloral hydrate, which is sometimes taken safely in large doses, and at other times it proves rapidly fatal, even in doses of ten grains. He inquired if this effect could be due to the coincidence of digestion and the change of chloral into chloroform by the alkaline bile, as Liebig believed.

Dr. Stockman, Edinburgh, Scotland, said that the amount of chloroform liberated from ten or fifteen grains of chloral hydrate would be so small as to be trifling. He thought that it was not decomposed, but that it acted as chloral hydrate. A good deal of dissatisfaction has been felt with chloral on account of its depressing action, and other agents such as hypnone and urethan, have lately been introduced to take its place.

Dr. H. G. Beyer, U. S. Navy, said that Mayer had explained the action of chloral hydrate as that of trichloracetic acid ; and, in fact, all other halogen derivatives of organic compounds produce their effects by the chlorine which they contain, which is said to be set free whenever these compounds are brought into an acid medium. For instance, the cells of the cerebrum are supposed to react slightly acid during intellectual activity, but when trichloracetate of sodium is injected into the blood, the chemical compound is decomposed, chlorine is set free, and sleep produced, or intellectual torpor, in the same manner as this is done by chloral hydrate.

Dr. John N. Uphen, of Richmond, Virginia, read a paper entitled

THE EMMENAGOGUE ACTION OF THE MANGANESE PREPARATIONS.

The usefulness of potassium permanganate when used as a deodorizer and disinfectant is based upon its readiness to part with its oxygen. Its mode of action is not clear, but in small doses internally it acts like iron, improves the general nutrition, and causes an increased and easier flow of blood at the menstrual period. The permanganate of potassium and the oxide of manganese are both used, the latter being more acceptable to the stomach than the former. It is given in gelatine-coated pills (gr. j. to ij. after meals), and to get its full effect it must be given before three successive periods. In amenorrhœa due to an impoverished and cachectic condition of the blood, oxide of manganese, given in combination with some form of iron, will undoubtedly prove of benefit. It is also useful where the condition is due to defective nervous or vascular supply when pain is present, due to fuctional disorder; also when *no* obstruction exists, but the endometrium is in a state of chronic congestion, or affected by inflammation due to obstruction which has been removed ; in all cases of vicarious menstruation ; in amenorrhœa of plethora and obesity ; *in fine, when the menstrual derangement is due to functional and not mechanical or obstructive cause.* Especially had he found it advantageous in membranous dysmenorrhœa. An interesting case was reported in illustration of the latter class.

Dr. DeWitt Clinton Wade, of Holly, Michigan, read a brief communication on a

FORMULA FOR OFFICINAL DILUTE HYDROBROMIC ACID (U. S. P.).

In 1874 he had first made the acid by the reaction of tartaric acid upon potassium bromide ; and in 1875 had published a paper recommending its use in therapeutics. It was made officinal in the last revision of the United States Pharmacopœia, but the product is very variable by the official process. He recommends the following for making a standard ten per centum.

SOLUTION OF HYDROBROMIC ACID.

Bromide of potassium, four (*Avoir*) ounces ; tartaric acid, five (*Avoir*) ounces ; water, seven (fluid) ounces. Dissolve the salt in the water and add the acid. When thoroughly mixed set aside in the cold for the precipitation of the acid tartrate of potassium. Decant the supernatant fluid and dilute with sixteen fluid ounces of water. The result is a ten per centum solution, by weight, of hydrobromic acid.

---

### SECTION ON ANATOMY.

### Fourth Day—Morning Session.

The first paper presented was entitled

THE FUNDAMENTAL ANATOMICO-MECHANICAL CONSIDERATIONS UNDERLYING THE SUCCESSFUL TREATMENT OF DEFORMITIES, DISEASE AND WEAKNESS OF THE SPINE, by Milton Josiah Roberts, M. D., etc., of New York, N.Y. He had began by saying that he had spared no time, labor, nor expense in studying this subject thoroughly, by testing all known methods, and felt that he could offer his results with some satisfaction to the Section. The corsets for the arrest of Pott's disease and other spinal troubles were made of a fine wire thread which was carefully woven over a plaster cast of the patient's body, so as to secure an exact adaptation. The great problems to be solved were —how and where to get hold of the body to support it without irritation, and how best to apply any remedies necessary. These problems, he thought, were simply mechanical. Bone should be the supporting point. He spoke of the great advantage of these wire corsets over the heavy and clumsy Plaster-of Paris jackets. He thought there was a great want of proper description of how to apply these apparatuses on the part of writers and lecturers. He mentioned, also, the ease with which these wire jackets followed the chest-walls during respiration. He had found no brace so good for the support of the head as the one that went over the head. He showed several specimens of Pott's disease and other spinal deformities, and also photographs, drawings and models.

Dr. William J. Herdmann, of Ann Arbor, Michigan, spoke of the great advantage of Dr. Roberts' wire jackets over the old plaster jackets, and insisted that such apyaratus could only be applied by one thoroughly interested in the subject and ingenious and skillful.

Dr. Max J. Stern, of Philadelphia, Pennsylvania, then presented a specimen of

AN ANOMALOUS MIDDLE THYROID ARTERY, the knowledge of which was of surgical importance in tracheotomy. He thought it was the first of its kind made public.

Dr. Herdmann had seen several such anomalies, and thought it not uncommon; but had never published his cases.

---

Afternoon Session.

Dr. W. X. Sudduth, of Philadelphia, Pennsylvania, then read a paper on

DEVELOPMENT OF BONE, in which, after defining the terms ossification-bone and osteoblast, he laid down as the result of his study of developing bone that the white blood-corpuscles were the basis of all the connective-tissue group. He emphasized the point that the osteoblast did not become calcified. In the process of calcification the secretion took place around and not in the cell, the calco-spherules joined, and solid bone was formed. This deposit of bone-salts took place in the interstitial protoplasm. As the ossification began, the first deposit of bone was at the periphery of the system, and as it approached the centre it was self-limiting. As calcification progressed the osteoblast decreased in size and its envelope grew thicker. He quoted several authorities and their definition of the different kinds of bone according to their development. His own definition of bone was that it was an aggregation or conglomeration of calco-spherules. His own division of bone according to its development was: (1) Interestitial; (2) intermembraneous; (3) subperiosteal; (4) intercartilaginous.

By interstitial bone he ment those bones which were not formed in cartilage and in which ossification took place prior to birth. The paper was illustrated by many microscopical specimens of developing bone, which were beautifully stained and mounted.

Dr. N. Stamm, of Fremont, Ohio, then read a paper entitled

ANATOMICAL POINTS OF VALUE IN THE DIAGNOSIS AND TREATMENT OF SOME OF THE JOINT AFFECTIONS.

He first described the anatomy of the synovial sac and proceeded to speak of some of the inflammatory and tubercular affections of the joints, confining his remarks to the knee and ankle-joints. He reviewed the etiology, pathology, and bibliography of the subject and then laid down the rules of treatment, the basis of which was rest and fixation.

Dr. Benjamin Lee, of Philadelphia, Pennsylvania, then read a paper entitled

CASE OF DEFORMITY OF THE SPINAL COLUMN PRODUCED BY MATERNAL IMPRESSION ON THE FŒTUS, in which he related a case of cyphosis supposed to have been caused by maternal impression.

In the discussion which followed, Dr. Joseph N. Dickson, of Pittsburg, Pennsylvania, and several others were of the opinion that many cases occurred in which the maternal impression had no visible effect upon the fœtus, and thought that if statistics of all such cases could be collected a small proportion would be found to be so affected.

---

### SECTION ON PATHOLOGY.

Fourth Day.

Dr. Leopold Servais, of Antwerp, Belgium, reported two successful cases of

REMOVAL OF THE SUPERIOR MAXILLA

for cancerous tumors.

Dr. Jackson, of Norfolk, Virginia, read a paper on

THE NATURAL AGENCIES EXHIBITING THE LIFE-PROCESSES OF PATHOLOGICAL ORGANISMS.

Professor N. S. Davis, Jr., of Chicago, read a paper on

CELLULAR PATHOLOGY—ITS UTILITY IN PATHOLOGICAL PROCESSES.

The author briefly referred to the views of Metchnikoff on intra-cellular digestion, and the illustrations of this process given by him and J. Bland Sutton.

He called attention to the fact that there is probably a cellular digestion in addition to the intra-cellular. By the former term he described the process which takes place when dead bone is removed or eroded by leucocytes, granulation-cells, etc. Under such circumstances, undoubtedly the living cells are the active factors in removing the bone, and there is no evidence of the occurrence of intra-cellular digestion. The removal is accomplished by a similar digestive process, but the cell acts by contact with the digestible body, not by first swallowing it. Digestion by cells in pathological processes was illustrated by the so-called absorption of extravasated blood, of fibrin in thrombi and in deposits, in croupous pneumonia, of cells killed by the exciters of inflammation of various kinds, of bone and various foreign bodies that are soluble in the living tissues but not in the liquids of the body.

Such powers of digestion were ascribed to the leucocytes and connective tissue cells and granulation cells, and probably to some tumor cells.

Metchnikoff's view, that leucocytes are the natural adversaries of micro-organisms, was referred to, but the assertion made that only in certain cases was it shown that leucocytes were inimical to bacteria. They are, however, the undoubted removers of dead micro-organisms, and probably by processes of intra-cellular digestion.

We possess no knowledge of the exact chemical changes that take place in these processes. Some facts are pointed to that show the predilection of leucocytes for peptone. The suggestion was made that possibly this was the form in which they required their nourishment.

---

### SECTION ON DISEASES OF CHILDREN.

---

### Fourth Day—Afternoon Session.

Professor Albert R. Leeds, of Hoboken, New Jersey, read a paper on

THE NUTRITION OF INFANTS.

He had undertaken to find a true basis for the preparation of artificial food by analyzing eighty samples of human milk. He found that human milk differs from cow's milk chiefly in the proportion and digestibility of the caseine, which is smaller in quantity and more easily digestible in human than in cow's milk. He belived that he had solved the problem by digesting the caseine by a peptogenic powder, easily obtainable and of constant strength, which, with the aid of heat, reduced the caseine in five minutes. Before this cooking, the milk had been first diluted with water in order to lessen the proportion of caseine, and then had been enriched by the addition of cream to restore the normal proportion of fat. The results of a very large number of trials, followed by careful observation, encouraged the belief that by this process the artificial feeding of infants had nearly reached perfection.

A paper by Professor D'Espine, of Geneva, Switzerland, entitled

OBSERVATIONS ON TRUE OR LOBAR PNEUMONIA IN CHILDREN, was then read by Dr. Cordes, of Geneva. The object of the paper was to maintain that there exists in children a special form of true pneumonia, which may be called central, from its location; congestive, from the violence of the inflammatory symptoms; and abortive, when its duration does not exceed two or three days. It is found in the interior of the upper lobe, and in its upper part. As the physical signs are detected with difficulty it may easily be mistaken for some disease which has the same train of general symptoms. The microscopic examination of the sputa reveals the presence of the micro-organisms commonly found associated with pneumonia.

A fatal termination is the exception, and when it occurs is generally the result of complications. The writer detailed cases in which death had been preceded in some cases by gangrene, and in others by gray hepatization. The best form of treatment is that which has for its object the reduction of the inflammation. Tepid baths, the compresses of Priessnitz, and similar methods of combating general and local heat are to be used. This treatment is alone reliable when there is high fever with cerebral symptoms.

Dr. Henry Ashby, of Manchester, England, then read a paper entitled

SCARLATINAL NEPHRITIS FROM A CLINICAL AND PATHOLOGICAL STANDPOINT.

The paper presented the results of his own observations in the wards and dead-house of Pendlebury Hospital for Sick Children, where fifteen hundred cases of the disease had been received in the past eight years. The disease has been observed in three forms: (1) An early or initial form, not especially important when compared with the others, and (2) a variety dependent on septic changes which leaves traces in the kidneys discoverable by the microscope. It occurs in the second and third weeks. In most cases this septic inflammation of the kidneys gives no symptoms distinguishable from those of general septicæmia. It does not extend so widely in the tissues of the kidneys as to impair their functions seriously. The urine is not diminished, and general œdema and uræmic symptoms are absent. (3) Post scarlatinal nephritis, or the nephritis of convalescence, far surpasses the other forms in interest and importance. It occurs in an apparently well or rapidly convalescing child, and is the last and often fatal assault of a disease which has been almost subdued. It occurs from the sixteenth to the twenty-fourth day. The kidneys, after this form of scarlatina, like the lungs in measles, are weakened by their efforts to elminate poison, and are more readily affected by fibrinous or croupous inflammation. In hospital practice, at least, cases of mild scarlatina are less liable than severe cases to be followed by nephritis, which ranges in severity from cases which might easily be overlooked to those in which the uræmic symptoms are sudden and alarming. The earliest and most characteristic signs are diminished urine and puffiness of the face, which may appear several days before albumen is detected. The amount of albumen and its specific gravity vary greatly in different cases. The urine continues to diminish in quantity, and œdema of the face sets in, with vomiting and other uræmic symptoms, when suddenly a crisis like that of pneumonia takes place, large quanties of smoky urine of low specific gravity passes, and the patient is convalescing again.

A paper on the

ANATOMICAL CHARACTERS OF SCARLATINAL NEPHRITIS, was then read by Dr. Frank Grauer of New York, N. Y.

The writer reviewed the post-mortem renal conditions in the initial catarrhal nephritis of scarlatina, and those which are found in the large flabby hæmorrhagic kidney, which belongs to septic inflammation. But he had more particularly studied post-scarlatinal or Klebs' acute glomerulo-nephritis, his observations being based on nine cases of this affection. The gross and microscopical appearances were described in detail. The kidneys were enlarged and hyperæmic, with the cortex unchanged or sometimes thickened and the glomeruli prominent. With a low power the glomeruli are larger than normal, and with a higher power apparently bloodless.

Although he had noticed swelling and proliferation of the glomerular epithelium he did not agree with the opinion of some that the capillary circulation is obstructed through compression of the capillaries by this proliferation, because in all the specimens which he had examined the loops of the capillaries were larger as a rule than normal, showing that the pressure was from within not from without.

He believed that the capillary obstruction is due to proliferation and thickening of the endothelial cells. The hypertrophy of the left ventricle, noticed in all his cases, was due (1) to the presence of some toxic element in the blood, and (2) to obstruction of the circulation in the Malpighian bodies, which compelled the left side of the heart to do more work.

He suggested that the term glomerulo-nephritis ought to be limited to those affections in which there is an obliteration of the loops of the capillaries in the Malpighian bodies, and not applied to those affections in which there is only proliferation and desquamation of the glomerular and capsular epithelium, which are common to all forms of chronic nephritis.

---

### SECTION ON OPHTHALMOLOGY.

### FOURTH DAY—MORNING SESSION.

At the opening of the session, Dr. X. Galezowski, of Paris, France, read a paper entitled

THE CURABILITY OF DETACHMENT OF THE RETINA.

He stated that the pathology of the disease is not entirely clear.

The writer had observed in twenty years, among 152,000 persons, 789 detachments, of which 87 were in both eyes, 63 in emmetropic and hypermetropic eyes, and 194 were traumatic; 13 occurred after extraction of cataract, 18 were syphilitic, and 4 in sympathetic affections. Tumor was found in 10 cases. Twice only he found detachment in retinitis albuminurica, although he frequently saw this affection. Cataract is very frequent in detachment of the retina.

Occasionally tearing of the retina is noticed, with the corpus vitreum introduced behind the retina, between the choroid and retina. He could say that the rupture of the retina is not so frequent, and considers it the consequence and not the cause of the detachment. Professor Von Graefe has said that the detachment is not curable, and the function of the retina is not restored. Dr. Galezowski had seen a case which showed alterations in the retina at the place of detachment, which had been completely cured. The patient gave the usual history of trouble with the vision, coming on suddenly and continuing for one or two or three months, with afterward recovery of sight. He explained the appearance of the fundus as seen by the ophthalmoscope, and illustrated them on the blackboard.

The conditions predisposing to detachment were said to be (1) choroiditis; (2) liquefaction of the corpus vitreum. In treating these cases he begins with antiphlogistic treatment, atropia, rest, etc., and he had in seven cases a complete cure—the retina completely adherent, and around the line of the separation atrophy, with pigment deposit and choroiditis disseminata. The first indication is antiphlogistics; to apply every month two, three, four, or five leeches, then atropine, and warm and cold compresses alternately, and in the intervals between the leeches he applies derivative plasters. Inside of five months he has completely cured detachment of the retina. Mercury and potassism iodide are also useful where exudation is present or in cases of constitutional disease.

Fifteen years ago he proposed iridectomy to stop the inflammation of the choroid, but it did no good in that way, although it stopped the iritis. Now he proposes a new operation. He considers the exudation behind the retina as being of the same character as the effusion in pleuritis or peritonitis, and has had an instrument which he exhibited made to aspirate the fluid. The instrument is a syringe with a stop-cock and an aspirating-needle. He introduces the needle through the sclerotic at a considerable distance behind the ciliary body, and passes it into the globe for some distance. Then he exhausts the air by drawing out the piston. If the needle-point is too far in, no fluid appears in the barrel of the syringe, but on withdrawing the needle gradually the fluid appears, and one, one and a half, and two gramms, is generally obtained. There is no inflammation after the operation. By this means two out of seventeen cases operated on were completely cured, and there is a certainty of amelioration in all cases.

In old cases he introduces a curved needle from behind forward through the sclera and the detached retina, before introducing the aspirating-needle, and when the fluid is drawn off a catgut ligature is drawn through as a seton and brought tight.

Dr. Abadie, of Paris, France, spoke on the causation of detachment in myopes, setting forth the gradual stretching of the sclerotic in staphyloma and its drawing away from the retina as a cause of the detachment, with remarks on treatment.

Professor P. D. Keyser, of Philadelphia, spoke of Von Graefe's method of tearing the retina through the detachment with a needle of De Wecker's trocar, and of the double-needle method. He had never been able to get a permanently good result, although the immediate effect was sometimes very pleasing. He thought well of Dr. Galezowski's instrument, but wished to know how it would act in old cases.

Mr. J. Richardson Cross, of Bristol, England, said that there was no doubt that the subject was an exceedingly difficult one. He had treated a case with diaphoretics, had pushed pilocarpine, and used the other commonly accepted means of relief, with no change till now. He had done sclerotomy in three eyes, two in one girl. One, in a woman, did no good.

Dr. E. Landolt, of Paris, France, recognized three kinds of detachments. The first is due to choroidal exudation; the second, detachment in myopia; and the third, traumatic detachments. For the first, the treatment is perfect rest and compress dressing. He quoted a case which was cured and had remained well for three years. In the second class surgical interference may be justifiable, because there is scarcely any hope of restoration of vision in the part affected. He illustrated by a diagram his mode of operating with a Graefe knife, and remarked on the possibility of sucking out vitreous if the syringe was employed. For the third variety, traumatic detachments, there were no general rules. Some are restored with simple rest, and others resist all treatment.

Professor E. Smith, of Detroit, Michigan, had been struck with the remarks of Doctor Abadie. In his first case he introduced an acupuncture needle, and the immediate result was brilliant; but the permanent improvement was *nil.* He preferred to do the operation suggested by Dr. Wolf, of Scotland, and would hope to get up adhesions to tie the retina down to the wound. Doctor Galezowski's operation looks rational, if he does not puncture the retina.

Dr. W. F. Holcombe, of New York, N. Y., remarked that he presumed Dr. Galezowski, when he spoke of a cure, probably did not mean complete restoration of vision, but only reduction of the detached portion, and its restoration to its original plane. He also described Sichel's operation.

Professor D. S. Reynolds, of Louisville, Kentucky, wished to know whether it were a fact in the eye as in other organs, that frequent tapping is followed by increased accumulation.

Professor A. W. Calhoun, of Atlanta, Georgia, wished to know what advantage is to be obtained by operating in cases of this kind of long standing. The eyes are blind, and vision is not restored, and the operation may set up inflammatory action. He had done sclerotomy many times, but without satisfaction.

Mr. H. Power, of London, England, found it difficult to understand how there could be restoration of vision in these cases of detachment. The pigment layer is detached from the retina and left behind, and does not re-attach itself again as before. He believed there might be an apparent restoration where the detached portion had hung down over the good part like a bag, and after reduction of the detachment there was an apparent improvement in that part of the retina.

Dr. Galezowski remarked, in closing, that he had only done the operation in bad cases. If it had been done in all cases, we might get a better percentage of favorable results. An antiseptic might be injected into the cavity. It is possible for the retina to resume its function of vision, but even if it were not, the arrest of the process and saving the rest of the vision is of value.

Dr. J. A. S. Grant (Bey), of Cairo, Egypt, then read a paper contributed by Dr. Brugsch (Bey), of Cairo, who was not able to be present, on

THE PREDISPOSITION TO GLAUCOMA.

He said that increased tension, the recognized test-symptom of glaucoma, might be produced in two ways, either by increased secretion, or by retention of the secretion, when normal in amount. He was inclined to think that the theory of retention was, at least in some cases, the one which appeared reasonable. It was a question whether eyes with small corneæ were more liable to be affected, and he gave some statistics which seemed to point in that direction.

The predisposition of the Semitic race to glaucoma is remarkable. In other races it occurs in the proportion of one to one hundred, here the proportion is four to one hundred. He had been surprised by finding children affected with glaucoma. The cornea of the pure Egyptian is decidedly smaller than that of other races, and he had been endeavoring to find whether the whole globe was also smaller, but his researches had been hindered by the trying climate, which produced decomposition in fresh eyes so rapidly. After iridectomy there appears to be a relaxation, and even an enlargement, of the corneal circle, as you can see if you notice that the coloboma will sometimes appreciably enlarge a considerable time after the operation.

---

AFTERNOON SESSION.

Professor A. G. Sinclair, of Memphis, Tennessee, reported

A CASE OF RETINAL GLIOMA ON BOTH SIDES.

A young child was brought to him with the history that some time before it was noticed not to see well, and soon appeared to be blind; then a white and somewhat lumpy appearance was observed in the pupil. Soon afterwards black lines were noticed over the surface of this appearance, the retinal vessels. On examining the eye Dr. Sinclair found congestion of the conjunctival and episcleral vessels, pupil slightly dilated, tension somewhat above normal. The same appearance was observed in both eyes, except that the left cornea appeared somewhat abraded in the center. Enucleation was proposed and was consented to. The right eye was removed together with about one half-inch of the optic nerve, and the left eye and the whole contents of the orbit, as far as possible without cauterization.

A careful and complete pathological examination of the right eye was made by Dr. T. Mitchell Prudden, who reported it as a *glio-sarcoma.*

The left eye and its appendages were examined by Dr. Carl Heitzmann, of New York, and his report gave the same conclusion. These two examinations and reports were made each without any knowledge of the other.

The case has now been observed for six years since the operation, and there appear to be no signs of the return of the disease, and the child is perfectly healthy. No heredity was discovered.

Professor Keyser, of Philadelphia, remarked that in cases of this sort he was doubtful if the child would live more than eighteen months or two years. One case of his had lived seven years, and one died in eighteen months after operation of glioma of the brain. He was doubtful if the successes were cases of true glioma.

Mr. Henry Power, of London, England, expressed the same doubt, although he has had some successes also.

Dr. Galezowski, of Paris, has operated for the removal of this disease, and one recovered and was afterward healthy. He had only seen four cases.

Professor D. S. Reynolds said that he thought it is a malignant proliferation conveyed through the lymph-channels. When it originates in the retina he saw no reason for its not being successful. He had had a case where he had enucleated both eyes, which were invaded simultaneously and equally. There was immunity for ten years.

*A propos* of Dr. Reynold's remarks as to the origin of glioma, whether in the optic nerve or retina, Dr. R. L. Randolph, of Baltimore, Maryland, remarked, that in a microscopic examination of a gliomatous eye very recently, he found the optic nerve-fibers pressed apart by the growth, which latter extended up to the severed end of the nerve. The whole optic nerve tissue was disorganized and, so to speak, monopolized by the growth. He inferred that the growth must have had its origin in the optic nerve.

Dr. H. C. Paddock, of New York, N. Y., read the next paper, entitled

ERGOT OF RYE IN OPHTHALAMIC PRACTICE.

No mention is made of this remedy in the earliest works on medicine, and even until very lately it has been considered to be a medicine for the obstetrician alone, and only to be good to stimulate contraction in the uterine muscle. The action of ergot is to promote contraction of the blood-vessels, as well in other organs and tissues as in the uterus, and especially in the blood-vessels of the eye. Soelberg Wells, in his work mentions it as a valuable remedy in congestions, episcleritis, etc., while Hammond says it possesses the property of contracting unstriped muscular fiber. It is certain that it does diminish the caliber of the vessels, and has a good effect in obstinate affections. It is a rational remedy, and nothing more is claimed for it than its general therapeutic properties. He gave a few cases in which it had seemed to exert a markedly beneficial effect.

In the case of a woman with conjunctival congestion and pain and discomfort, the whole trouble was stopped on the third day. In still another case, twenty-five years of age, there was conjunctivitis, iritis, retinitis, and ciliary neuralgia. Under ordinary treatment she improved for four or five weeks and then came to a stand still. She was then given ergot, and was discharged cured in a few weeks. In regard to the use of ergot, he begged to remark that it is a reliable drug, that it must be used in maximum doses and for several days, and that it is a good tonic; that atropia should be used in iritis, and reliance on general treatment for any complication that may arise, was advocated.

Mr. Henry Power, of London, England, then read a paper written by Mr. P. H. Mules, of Manchester, England, who was unavoidably absent, entitled

EVISCERATION AND THE ARTIFICIAL VITREOUS.

The object of the paper was stated to be to lay before the section the results of an operation which was intended to produce an improved appearance over the result in evisceration alone, with a clean cavity free from muco-pus. The mode of shrinkage after evisceration was discussed, and a blackboard demonstration of the method of operation given. The incision is made through the conjunctiva around the cornea, elliptical with its long axis horizontal. The conjunctiva is dissected back for a short distance, and an elliptical incision made through the sclera of about the same size and in the same position as that in the conjunctiva. The contents of the globe are then carefully cleared out, which is easily determined by the view of the white sclerotic in the interior of the eye. The glass ball is then introduced, the edges of the scleral wound carefully fitted together over the ball, care being taken to have no tension at the point of union, united by sutures, and the conjunctiva then brought together and sutured. There may be considerable reaction and some pain, which is treated in the usual manner. Mr. Power had done the operation about a dozen times, and had had three bad results. One suppurated, and in two the wound yielded and the glass globe was pushed out. Catgut sutures are used.

Mr. Cross, of Bristol, England, thought Mr. Mules' operation would be a permanent one. The shield over such a globe has a better motion than any artificial eye on a natural stump can have, because the muscles have been placed in a position more nearly corresponding to that in the natural eye. It does not make any difference whether one use a glass globe or a silver, or, as has been lately, a celluloid one. An advantage of this operation is that the lower lid is preserved, and is not gradually obliterated so that the shell cannot be retained. It cannot be said that Mules' operation will prevent sympathetic ophthalmia, because we cannot entirely clean out the globe. There may be affection of the lymphatics exterior to the globe already before evisceration, and it is, of course, impossible to reach them in this operation. Mules' operation is not to be compared with enucleation in sympathetic ophthalmia. We operate in sympathetic ophthalmia to save the other eye. It is not a question of appearance, but of sight.

Dr. Galezowski, of Paris, France, would divide the question into two parts—evisceration and the introduction of the glass ball. He saw this first fourteen years ago, with Professor Richet, in Paris. Great inflammation resulted, and it was not possible to introduce the glass ball. Enucleation was done six months after and the patient got well. He saw another case four months ago. Six months previous a good operation had been done, but a sinus had formed in the region of the wound. Treatment with antiphlogistics, antiseptics, etc., was of no avail. Enucleation was done and all was well. One year is not long enough for a final conclusion in the matter of result. In sympathetic ophthalmia there must be danger if we leave the smallest piece in the eye, and the enucleation must be completely and carefully performed, so as to leave none of the sclerotic attached to the nerve.

Dr. Baker, of Cleveland, Ohio, as an illustration of the ability to retain a foreign body in the eyeball with impunity, related a case which had occurred to him. A man had his eye burned severely with sulphuric acid, so that an unsightly ball was left, with no cornea. He put in a glass button like a collar-button, and the man had been wearing it comfortably ever since.

Professor Keyser, of Philadelphia, Pennsylvania, stated that he would try Dr. Mules' method. He had had very severe inflammation occur after evisceration. To illustrate the danger of sympathetic ophthalmia, when enucleation is not effectually performed, he related a case where a small button of sclerotic was left on the divided nerve. He advised to get it all out, but it was not thought worth while, and sympathetic ophthalmia set in. He believed it was better to do evisceration early.

Dr. Dibble, of St. Louis, Missouri, had seen two cases of bony deposit inside the sclerotic, and believed that in such a case the pressure of the glass ball would produce irritation and perhaps sympathetic trouble.

Professor E. Smith, of Detroit, Michigan, had seen Dr. Power do one of these operations, and and although Dr. Power had predicted considerable chemosis and severe reaction, there was no trouble whatever, and a happy healing resulted. There was not a bad symptom. He laid stress upon the fact that the edges of the cut sclerotic should just meet, not overlap or strain.

Dr. R. L. Randolph, of Baltimore, Maryland, read a paper contributed by Dr. H. Gifford, of Omaha, Nebraska, entitled

FURTHER CONTRIBUTIONS TO THE STUDY OF SYMPATHETIC OPHTHALMIA.

It related to the posterior lymph-stream, and detailed the results of experiments with injections of India-ink and anthrax. The course of the injections was ascertained by examining the eyes at periods after injection, and it was found that, even in neurectomized eyes, the coloring matters or anthrax bacilli had reached the brain from the posterior chamber; even where the cerebral end of the cut nerve contained none, showing that there is a current from the posterior part of the eye back to the brain, independent of the channels of the optic nerves.

Mr. J. Richardson Cross, of Bristol, England, next contributed a paper entitled

RETINOSCOPY; IT PROMISES A RAPID AND RELIABLE METHOD OF ESTIMATING ERRORS OF REFRACTION, AND IS A TEST OF THE GREATEST PRACTICAL VALUE

He stated that the full correction of the refractive error is not the one that is always most likely to be accepted by the patient, when determined by the upright or direct image method. With trial glasses it is often necessary to use atropine, but in retinoscopy he has only found it necessary to use atropine in cases of spasms of accommodation. In all other cases cocaine is quite sufficient. Either the plane or the concave mirror may be used. The production of the image at the far point of the eye in myopia is the important unit in the question of retinoscopy. The shadow observed in myopia is that of the erect image projected from the observed eye. With illumination, no movement, and no shadow, the observer must be near the conjugate focus of the rays. It is a usual thing to have one constant point to calculate from and work from, and the usual standard of a little over a metre is objectionable, because it is necessary to get up and approach the patient to make every change of glasses. Dr. Cross could use an optometer made by Doyne, which he exhibited, at a distance of eighty centimetres, which obviated the necessity of moving with each change of the patient's glass. Mr. Cooper has brought out a somewhat more complicated optometer, by which you are enabled to sit at the full distance and turn the disk.

The proper point to estimate the refraction in the patient's eye is the macula, but it is not easy; the disk is easier, but not so accurate on account of the occurrence of the physiological cup so often. The writer preferred to take a point midway between the two, which could easily be done by making the patient look at the other side of the face.

The results of retinoscopy are quite satisfactory; unsatisfactory in only about ten per centum.

The differences between the result with and without atropia are slight in degree, in a spherical direction, and do not exist as to the cylindrical correction. A much higher degree of latent hypermetropia is uncovered by retinoscopy than by trial lenses.

Professors Keyser and Reynolds rose to questions of information as to the abolition of the accommodation in the use of the method, and

Dr. Galezowski stated that he liked the method, but did not like the name. He believed the shadow to be produced by the changes in the cornea, and that the proper name is keratoscopy, An advantage is that one can make a diagnosis of the reflection without atropia.

Dr. Galezowski's assistant, Dr. Parent, had described it very accurately, and used the name keratoscopy. In proof of its being the shadow in the cornea, the best diagnosis of staphyloma of the cornea could be made by this method.

There is no reason at all for the term pupiloscopy.

Dr. A. R. Baker, of Cleveland, Ohio, also read a paper on

RETINOSCOPY.

Although retinoscopy has been extensively employed by general practitioners and specialists in England and France, it has been almost entirely ignored by German and American practitioners. While many competent observers have written extolling the method, no less an authority than Professor Hirschberg, of Berlin, remarked in a laughing manner, when the writer asked his opinion of the value of retinoscopy, "Oh, that is a lazy English method, and don't amount to much." And Dr. Loring, in his excellent work, thinks that it does "nothing which cannot be more easily and more expeditiously performed by the upright image."

Probably one reason for the rejection of this method by Professor Hirschberg and Dr. Loring, is their extreme accuracy in the use of the ophthalmoscope. But this accuracy was, he believed, limited to only a very few, and it required a large amount of clinical material to become proficient, and few, if any, master the ophthalmoscope sufficiently under forty years of age to become proficient.

In teaching the method it is better to select an emmetropic eye for the first, and dilate the pupil. A concave is more convenient than a plane mirror. If the plane mirror is used it is to be remembered that the image will move against the mirror in myopia and with it in hypermetropia, and the reverse with a concave mirror.

Professor S. M. Burnett, of Washington, D. C., proposed the name scioscopy. He prefers the plane mirror.

Professor A. W. Calhoun, of Atlanta, Georgia, wished to know if all cases were corrected by this method so that they did not come back dissatisfied.

Mr. Cross, in closing, stated that retinoscopy was a name in general use now, and therefore it was better not to change it. He thought he would continue to use it. It certainly was not dependent on the view of the cornea, and he certainly never should call it keratoscopy.

---

### SECTION ON OTOLOGY.

### Fourth Day.

Professor E. DeRossi, of Rome, Italy, read a paper on

RESEARCHES ON THE MICRO-ORGANISMS IN THE EUSTACHIAN TUBE OF HEALTHY INDIVIDUALS.

When it was established that there existed in the saliva and in the follicles of the tonsils of healthy men indisputable evidence of the presence of pathogenic micro-organisms, and especially of the presence of the coccus pyogeneus aureus, it seemed to me desirable to undertake certain experiments to ascertain whether or not there existed micro-organisms in the eustachian tube; and if so, to ascertain their peculiarities. I fully realized the difficulties of obtaining positive and indisputable results. I assure you, however, that I have taken all the necessary precautions; that I have observed all the indispensable minutiæ which present scientific observations exact.

The method of procedure has been as follows: A fine platinum wire was fastened into a silver catheter. This wire was of such a length that it could be projected from the tubal end of the eustachian catheter to the distance of one centimetre. The terminal end of the platinum wire was bent so as to form a loop in order to retain a sufficient quantity of the mucus collected from the inside of the eustachian tube.

Before sterilizing the instrument with the usual care the wire was withdrawn into the interior of the sound, then the tubal extremity was sealed with a thin coating of celloidine and every catheter so prepared was placed in the sterilizing tube.

In introducing the catheter it was passed as far as possible into the mouth of the eustachian tube, and by pressing on the proximal end of the platinum wire the thin coating of celloidine was broken and the looped end of the platinum wire projected to the distance previously determined. It was then withdrawn to the inside of the catheter. The loop of the platinum was immediately employed for the culture plates of gelatine, and later the colonies which were developed were cultivated in tubes.

The experiments were begun on the 12th of April, with the assistance of Professor Guarner, of the Institution of Pathological Anatomy in Rome. In that institution, through the kindness of Professor Marchiafava, all the necessary appliances were at our service.

The mucus was taken from the eustachian tubes of twelve persons at different times between the 12th and 30th of April. From each loop of wire two attenuations were used for plate culture. Positive results were obtained only from six of the cases—six out of twelve.

Case 2 gave two successful cultivations. The planting took place on the 22d of April, and by the 1st of May the colonies were developed. In the plantings from cases 1, 7, 8, 9, and 10 the colonies were well developed on the 3d of May. The plates belonging to the other cases remained free from organisms.

The examination of the colonies developed presented the following features:

Plate No. 2 in the first attenuation, the colonies were of a yellow color, and the surrounding gelatine was fluid. Examined under the microscope it revealed the streptococcus. Further from both the first and second cultivations a colony which was of a white color was developed which revealed under the microscope the grape form, staphylococus.

Plate 1 exhibited two colonies, one gray, the gelatine remaining solid; the other white, the gelatine liquifying. Under the microscope the white colony revealed torules; and the gray colony, with the gelatine unchanged, gave the streptococcus.

Plate 7 presented a round colony of a yellow color, the gelatine fluid. The microscope demonstrated the presence of short rods (bacilli) of a length varying from 0 mm to 1 mm and 0.5 mm thick.

Plate 8 gave two colonies, one round and of a yellow color; the other yellow, but irregular in form. In neither of these cases did the gelatine become fluid. The microscope revealed a staphyloccus from 1 to 1.5 mm long.

Plate 9 revealed a colony irregular in form and liquid gelatine. It was composed of filaments of different lengths; some from 10 to 12 mm, and others 1 mm long. The thickness varying from 0.5 to 0.7 mm.

Plate 10 revealed a colony of a yellow color and the surrounding gelatine fluid. It was composed of rods (bacilli) from 1 to 3 mm long and from 0.6 to 0.8 mm thick.

From all these colonies pure cultures were made in gelatine tubes and their identity established after the culture. Cultures were also made on potatoes; and finally from both the cultures in gelatine and the potato, transplantations were made to animal tissues.

It would be useless here to detail the individual cases. Experiments were made in the peritoneum and subcutaneous tissue in the rabbit and guinea pig, but in no case was any pathological development manifest. We are not to infer from this that in the mucus of the eustachian tube there never exists pathogenic organisms. It is necessary to investigate, and I present to the congress these notes as an interesting study worthy of being pursued.

Professor G. E. Frothingham said he had listened to Professor De Rossi's very interesting paper, presenting the result of his original researches in a field that had not been before investigated in this manner. The result of the investigations is such as confirms views he had for some time held from simple empirical observation. He had already expressed these views, and his reasons for them, and would not now repeat When he had declared these views, he had no knowledge of any investigation in this direction. It is peculiarly gratifying, when scientific discovery harmonizes with facts learned, empirically, as did Koch's discovery, that cholera bacilli cannot live in acid solutions, harmonize with the fact observed years ago, in the Pennsylvania Almshouse, that the use of sulphuric acid lemonade would sometimes prevent the spread of epidemic cholera. We cannot say the result is negative, because Professor De Rossi discovered no pathogenic organism. He thinks the presence of these organisms in great numbers, whether pathogenic or not, serves to irritate the membrane, and, under certain conditions of atmospheric change, and of the constitution of the patient, will lead to inflammatory action, acute or chronic. We should not forget this effect, since, in respiration of contaminated air, such vast numbers of germs may be lodged upon the nasopharyngeal spaces, and thus reach the ear.

If it be urged that *these* are not pathogenic, who can say under what circumstances they may not become so? The virus of syphilis, or gonorrhœa, is not pathogenic for some individuals; exerimentors have bathed their eyes for hours with the secretion of Egyptian ophthalmia, and after exposure to wind and dust, for some hours afterwards, had suffered no attack. He thinks no one now knows nearly all the conditions that determine the question, as to whether certain of these germs are always harmless. Investigation must be made to decide this.

As the etiology of certain chronic diseases of the middle ear are so obscure, and the treatment of them so unsatisfactory, that it constitutes a reproach to otology, it becomes us to hail with welcome every carefully conducted scientific investigation in this direction. Professor De Rossi is a pioneer, has done good work, and is entitled to our thanks.

Dr. R. Tilley thinks it interesting to observe how different listeners get consolation and encouragement. While the previous speaker derived encouragement from the paper read in support of his views of the pathological influence of these organisms, I, on the other hand, derived encouragement from the paper in support of the idea, that the influence of certain organisms is, at present, greatly over-estimated. I am glad the observations have been made and reported to us, notwithstanding their negative results, even after the germs were introduced into the peritoneal cavity and into the subcutaneous tissue.

Dr. A. B. Thrasher, Cincinnati, Ohio. I am much pleased to learn of the admirable researches of Professor De Rossi in this direction. That he has found bacteria in this region only accentuates what we have heretofore pretty well known, viz.: that all mucous membranes in contact with the external air contain bacteria. But these micro-organisms are by no means all pathogenetic. It appears to me that we are especially indebted to Professor De Rossi for his endeavors to discover which are the pathogenetic germs in this region. When the pathogenetic bacteria, or bacilli, have been certainly recognized, then can we say that we have made some satisfactory advance in this direction.

Dr. H. Gradle, Chicago, Illinois, remarked that the admirable researches of Professor De Rossi showed us an analogy between the eustachian tube and other mucous passages like the fallopian tubes, in which Fraenkel had found several pathogenic micro-organisms; or the mouth, in which numerous, harmless and virulent bacteria are known to exist. In otology it is necessary to distinguish accurately between exciting and predisposing conditions. The only exciting causes of suppuration are some micro-organisms, and occasionally chemical irritants.

If "taking cold," that is to say, temporary foreign circulatory disturbances, be accounted a cause of sudden inflammation of the middle ear, it is really an influence only which permits the bacteria, already present in the tube, but yet outside of the tissues, to multiply and enter the tissues and produce disease. Similarly, the presence of these bacteria in the tube explain why a nasal douche can, at times, produce acute inflammation of the middle ear, by conveying infectious material into the ear. Water, alone, could not give rise to such consequences.

Dr. Leartus Connor, Detroit, Michigan, stated that he is pleased to know a new method for the study of aural diseases. In normal conditions, we find that the bacteria cause no harm; it becomes important to study bacteria under such additional conditions as result in actual disease, and then we shall have light of added value.

Professor De Rossi, in closing, said "while the experiments I have narrated gave negative results, yet it is quite possible that under the depressing influence of cold draughts, or other causes, these organisms may contribute the determining influence which may result in some of those outbursts of inflammatory trouble, which sometimes come on so unexpectedly."

Dr. S. O. Richey, of Washington, D. C., then read a paper, entitled:

IS GENERAL ATROPHY OF THE CONDUCTING APPARATUS OF THE EAR IDENTICAL WITH PROGRESSIVE ARTHRITIS DEFORMANS?

The name General Atrophy of the Conducting Apparatus may not be better than the numerous other names by which this affection is designated, but it has the merit of describing the the result of the process as we see it, instead of indicating it by some particularity of its course. Some attempt will be made to show its probable neurotic origin in the spinal system by its similarity to a more general affection, which has been supposed to find its source there.

Atrophic degeneration of the conducting apparatus of the ear may not be, to any great extent, inflammatory in any part of its course is *not* "pre-eminently local in its character" *is* influenced by constitutional dyscrasia, probably begins at the cervico-spinal nervous centres, and is propogated through the sympathetic nervous system, or the sensory spinal nerves, interfering with local trophic action.

Many pathological changes have been observed in the cavity of the middle ear at its examination after death, but we are not assured thereby that any given structural variation has been a result of this affection alone, as we are denied the opportunity of observing it during the progress of the disease. For many reasons, we must think it has a broader pathogenesis than that usually accredited to it, in the exposition of which we may be aided by the processes of analogy and induction.

Garrod says of Progressive Arthritis Deformans (Reynold's System of Medicine, Vol. 1, p. 555). "It is much easier to prove what rheuma-"toid arthritis is *not*, than to give the slightest "clue to what it is. * * * * ; it appears to "result from a peculiar form of malnutrition of "the joint textures, *an inflammatory action with* "defective power * * * * ; it usually oc-"curs in weakened subjects, and exposure to "cold in many cases is the exciting cause of its "development." Weber (Jour., M. & N. Dis., Vol. VIII, 1883, p, 630) considers it of neurotic origin. In its entire history, except in the functional peculiarities of the locality attacked, it is almost a complete analogue of atrophy of the middle ear; in causation, symptoms, progress, and therapeutics. It would be an advantage to study the process as we can not in the ear.

Herewith is a parallel of the two affections:

| PROGRESSIVE ARTHRITIS DEFORMANS. | ATROPHY OF THE CONDUCTING APPARATUS. |
|---|---|
| (1) It is seldom fatal. | (1) We do not know it ever to be fatal. |
| (2) At an early stage swelling and the appearances of ordinary inflammation are prominent. | (2) At an early stage, this may be the cause of symptoms of inflammation. |

| | |
|---|---|
| (3) When the effusion into the joint is absorbed the capsule is commonly found thickened, the cartilages are sometimes absorbed and the ligaments so much lengthened as to allow unusual mobility and dislocation. | (3) When this happens it would be liable to cause tinnitus, or impaired hearing, or both; a flapping mt., and disarticulation of the ossicula. |
| (4) At the commencement of the process, slow absorption of the cartilages takes place, often followed by fatty degeneration, and the formation of ligamentous bands. | (4) The result of this change has been seen in anchylosis of the ossicles, especially of the stapes; retraction of the mt., and bands of adhesion in the cavity. |
| (5) Heredity does not seem to influence the affection, for one member of a family may be affected, and the rest be free. | (5) Complete correspondence. |
| (6) Is frequent among women, and rare among men. | (6) Is more frequent among women than among men. |
| (7) It occurs at any age, and individuals of weak frames whose extremities are cold are most liable to the disease. | (7) The symptoms are manifest at middle age, or just before, and at any later period. It may begin in the earache of children; cold extremities are common. |
| (8) Everything debilitating, as uterine hæmorrhages, prolonged grief, persistent mental distress, loss of rest and dissipation, damp dwellings, poor food, and all rheumatic influences are supposed active causes. | (8) Idem. |
| (9) By test, no uric acid, or urate of soda, *thus removing rheumatism* and *gout* from consideration as causes; reduction of phosphoric acid in the urine. | (9) No tests, so far as I know. |
| (10) The disease is slowly but steadily progressive. It may be stationary for a time, but exacerbations are sure to follow (Weber). There is slight remission, but no intermission during the rest of the patient's life (Haygarth). | (10) There are long intermissions in progress, judged by the impairment of function. |
| (11) It usually begins as a subacute disease. | (11) An open question. |
| (12) It is very intractable. When the disease is not advanced, *the affected joints few in number*, and progress slow, the prospect is more hopeful, especially if there is no disease to keep up the impairment of the general health. | (12) Idem. |
| (13) there is generally aching of the affected joints prophetic of an increase of pressure in the atmosphere. | (13) Any existing impairment of hearing, or tinnitus is increased under the same circumstances. |
| (14) Frequent mental depression without a known sufficient cause. | (14) Idem. |
| (15) It does not lead to suppuration, but to atrophy and more or less deformity. | (15) Idem. |
| (16) The treatment must be sustaining. Local treatment by blisters, iodine paint, and croton oil in the beginning. Later use counter-irritation; later still, friction and slight motion. Living in a moderate winter climate nutritious food, warm cloth ing, etc. | (16) This general line of treatment is the best with which we are acquainted. |

Arthritis deformans begins in the smaller joints of the body, is symmetrical in appearance and progress with lesions of the tissues surrounding the joints, atrophy of the muscular tissue, and in old cases a state of fatty and connective tissue degeneration (Weber). The Lilliputian joints of the ossicula auditus are peculiarly exposed to atmospheric changes by their location, and are in one of the extremities of the body, for which reasons they would seem to be more liable to an attack of this affection than even the joints of the head or foot. Rheumatoid arthritis, beginning in the small joints of the extremities, advances to the larger joints of the body, in which fact we may find an explanation of the pressure and pain about the head, and the diminution of intellectual apprehension, so common in cases of profound deafness in advanced aural atrophy. It may furnish a better demonstration of the deafness of boiler-makers, ship-caulkers, and locomotive engineers. Taking its symmetrical onset and advance as a point in evidence of its neurotic origin, it may also explain the change in voice so commonly met with among the profoundly deaf, who have become so by slow and progressive stages, for the recurrent laryngeal nerve makes the connection between the cerebro-spinal nerve centers and the vocal chords very intimate. The recurrent laryngeal is supposed to get its motor power from the pneumogastric, and irritation of the pneumogastric in the upper part of the neck has been proven by experiment to cause heat and tingling of the ear. Jewell (Jour. M. and N. Dis. 1874, 426) "looks upon articular rheumatism, as well as certain painful affections of the joints simulating rheumatism, as produced * * * by disease of the nerve trunks or nerve centers, leading to decided local irritation at the peripheral termination of certain nerves," and Brown-Sequard has shown that nerve fibers going to the blood vessels of the various parts of the head, come out chiefly from the spinal cord by the roots of the last cervical and the first dorsal nerves.

Arthritis deformans, nervous exhaustion, and aural atrophy (progressive deafness) very greatly resemble each other. Each follows causes exhaustive in character; does not terminate fatally; most of the symptoms are subjective and functional, and often unaccompanied with apparent structural variation. In each there is periodical hopelessness and discouragement. In nervous exhaustion and the ear affection there is diminished ability to fix thought on any subject (lack of mental control) and change in the voice; and Garrod claims that the irregular form of arthritis sometimes attacks the internal (middle?) ear and the larynx, and causes hoarseness and a peculiar dry cough.

In the deafness of boiler-makers, undisturbed control of equilibrium and the absence of vertigo argue against a theory of labyrinthine trouble. Buck (N. Y. Med. Rec., July 5, 1875) thinks the peculiarities of these cases due to rigidity of the ligament at the base of the stapes or to some change in the membrana secondaria, which to my mind is the most natural explanation.

Arthritis deformans may occur at almost any age; at first in the most exercised small joints, and if neglected it will progressively attack every joint in the body. It would rarely be recognized in the ear before the age of thirty, when the true function of the ear begins to be impaired in the late stage of atrophy, though it might have existed from the age of four or five years, at which time it would have been in its inflammatory stage.

Even in childhood a differential diagnosis might be made from the catarrhal affection, and we may reason that the disease at the foundation of the atrophic process may *begin at any age*, although the atrophy is a medium senilis.

Von Trolsch thought the disease without catarrhal symptoms should be given a different classification, but it is yet generally classed as a catarrh by authorities, though Pomeroy (Dis. of the Ear, p. 148) in a cursory way says: "I believe that the rheumatic diathesis, in many instances, has much to do with the obstinate character of this affection; the rheumatic inflammation, according to its well known predilection for fibrous tissues, finding a lodgment in the muco-periosteal lining of the drum."

Whether or not atrophy of the middle ear is of the same origin as arthritis deformans, it has a more extensive pathology than that allowed to it.

Dr. C. M. Hobby, Iowa City, Iowa.—I can recall seven cases of arthritis deformans in persons related to each other not more remotely than second cousins, and occurring in the ramifications of a large family, and among more than thirty members of this family that I can now recall, there has been but one case of deafness.

Dr. L. Turnbull, Philadelphia, Pennsylvania, saw a case of this disease, arthritis deformans, on Canonicut Island this summer, every one of whose joints was immovable; he was like a chalk man. When I called to treat him for ulceration of the cornea his hearing was perfect. In another case that I saw every joint was out of its natural position; the hearing was perfect. (Inquiry being made, whether these gentlemen had examined the ears, and if they found any structural change; they answered, they *had not examined* the ears.)

Professor G. E. Frothingham, Ann Arbor, Michigan, said he had listened to Dr. Richey's paper with much interest, and all would admit that he had presented the theory with sufficient show of argument to challenge attention. While Professor Frothingham could not say that he is ready to accept the views presented, they are worthy of more careful consideration than could be given in an off-hand discussion of the subject, which, in the aspect in which Dr. Richey presented it, is new at any rate to him. It is true we have long acknowledged certain changes in the drum-membrane and the articulation of the bones of the ear, as due to a gouty or rheumatic condition, but the claims made in the paper he has not met with before. It is a subject upon which he must reserve his opinion until further consideration, as it does not fully accord with his present views. He is all the more glad on that account to hear this view presented, as progress is best made by interchange of views, and stimulation to research grows out of opposing theories. He will repeat "that man is a public benefactor who makes *two* blades of grass grow where one grew before." On the same principle, that man is a medical benefactor, who gives us two ideas where we have but one before.

In obscure subjects, like the disease under consideration, we should take into respectful consideration any plausible theory, and, for one, he is willing to devote to it careful study.

Professor E. De Rossi, Rome, Italy, inquired in what way Dr. Richey would distinguish between atrophy, due to the cause suggested by his paper, and atrophy of the ear consecutive to hypertrophic processes.

Dr. Richey stated that in the published abstract of his paper he had stated that the question of atrophy of the ear, consequent to hypertrophy, would not be raised in the paper. In answer to

Professor De Rossi, however, he said the *history* of the case would suggest an antecedent hypertrophic condition, by increased secretion and diminution of the upper air-passages at some time; periods of greatly impaired function with relief, often without foreign agency; a subacute catarrhal condition, with thickening of the membrane involved, due to passive congestion, the impaired hearing being noticeable, especially during the exacerbations; and the absence of neuralgic pain. In atrophy, following arthritis deformans, on the other hand, there is, if any, very slight thickening of tissue; no perceptible increase in secretion; very slow, and progressive impairment of hearing, intractable in character; and neuralgic pain.

He did not anticipate immediate acceptance of the views offered. How can anchylosis, with prominence of the malleus and incus, be better explained? Is *dry* catarrh an inflammation? Mr. Henry Powers, of London, England, in his remarks upon bacteria in the section on ophthalmology, had intimated that the germs might, by migration, produce the joint affection, and Dr. Richey could not see that such a supposition would invalidate the theory of a neurosis, but, on the contrary, would do much to support it. He believes the bacterial theory, in explanation of this obscure affection, to be the *other* idea in Professor Frothingham's mind.

As arthritis deformans begins in the smallest and most used joints in the body, it might exist in the ossicula auditus, and be absent elsewhere. Drs. Turnbull and Hobby had not examined the structures of the ears in the subjects mentioned by them; they *did not notice impairment of hearing.* Their observations are therefore negative, for, how often do we see distortion of the malleo-incudal articulation, without perceptible impairment of hearing.

Dr. C. M. Hobby, of Iowa City, Iowa, read a paper on

CEREBRO-SPINAL FEVER AS A CAUSE OF DEAFNESS.

Cerebro-spinal fever occurs very early in life, fourteen per centum of the cases of deaf-mutism thus caused having originated under one year of age among those personally examined, fifty-seven per centum originating under two years of age, one case noted at two months and five at three months. What would be the percentage if the proper value were given to the record "born deaf?"

Investigation has heretofore been mainly directed to consanguinity and prenatal influences as causes for deaf-mutism, and based upon the startling, apparent, proportion of congenital cases. Reports of from thirty to sixty per centum of cases "born deaf" are acknowledged by many superintendents to be valueless; probably ten per centum will more than cover the congenital cases.

The nasal diseases of infancy are not so operative as has been supposed, a large proportion of so-called congenital cases being due to early intracranial disease.

Diagnosis of cerebro-spinal fever in infancy presents many difficulties. Cases of deafness attributed to scarlet fever have been found without tympanic lesions, and without ability to perceive sound either aerially or by bone-conduction.

In eighty-four deaf-mutes, where the deafness was caused by cerebro-spinal fever, sixty-three per centum had absolute loss of hearing in both ears; in about the same percentage no lesion was found in the tympanum by inspection of the membrane.

Blindness after cerebro-spinal fever is usually caused by suppurative choroiditis, but atrophy of the optic nerve, without ocular lesion, occurs (two cases reported). Deafness probably due in the vast majority of cases to disease of the labyrinth, but occasional lesion of the auditory nerve is probable; coincident paralysis of the facial is at least very rare; sense of taste is sometimes lost or affected. Blindness resulting at the same time with deafness is not very uncommon.

From the foregoing considerations, and from the comparison of the annexed tables, with each other, and their interpretation in the light of general observation and experience, I think the following conclusions are warranted:

1st. Cerebro-spinal Fever is prevalent throughout the United States, and is a constant factor in the production of deafness.

2d. That, upon the surface, it appears equal in importance as a cause for total deafness with Scarlet Fever.

3d. That when proper weight is given to the reports of causes of deaf-mutism, Cerebro-spinal Fever is the disease, above all others, producing total deafness, which the non-professional mind would class as "Fever," "Congenital," or "Unknown."

4th. That from reasons, some of which are specified above, and others of which are apparent to any physician who has studied the defective classes, it is probable that not more than ten per cent. of the cases of deaf-mutism are actually congenital.

5th. That with this assumption there appears upon the face of the reports thirty-five per cent.

of cases of total deafness, for which no rational explanation is offered.

6th. That the greater part of this thirty-five per cent. result from intra-cranial causes, including under intra-cranial disease, affections of the labyrinth.

7th. That it is important that more thorough investigation of Cerebro-spinal Fever be made, and especially of its relation to deaf-mutism, with the hope, in the light of more perfect knowledge of its cause and methods of dissemination, the production of deafness from this cause, may be diminished, as much as the sources of blindness were cut off by vaccination.

Dr. L. Turnbull, Philadelphia, Pennsylvania, inquired if Dr. Hobby had employed the hearing-trumpet in testing his cases of deaf-mutism. Are there not epidemics of meningitis in the western countries? Has not syphilis an important agency in the production of deaf-mutism in young children?

Professor G. E. Frothingham said his examination of the cases of deafness produced by Cerebro-spinal Fever agreed with that of Dr. Hobby, as stated in his paper. He had found, in a large majority of cases, a normal appearance of the drum-membrane. He had met with mixed cases, of which the history pointed to cerebro-spinal meningitis so positively, that there could be no doubt that the patient had suffered from it; there had also been inflammation of the middle ear, and, in such cases, the pharynx and nasal cavities had shown an inflammatory condition; while these had been much benefited by treatment, those, presenting normal drum-membranes with pervious eustachian tubes, he had found to be hopeless. He had heard of improvement in one instance only; the family of a child informed him that it had partially recovered its hearing. He had once seen an infant with double optic atrophy from cerebro-spinal meningitis. For three or four months its condition was such that its attention could not be attracted by light, while the optic papillae were glistening white. It, afterward, recovered sufficient vision to reach for large objects, and seemed to delight in viewing them. These cases could be explained by supposing that the destructive change had not involved *all* the nerve cells and fibres, in the rapidly growing child, but had left a small number capable of future growth.

Professor E. De Rossi inquired whether Dr. Hobby had made *post mortem* examinations in any of the cases?

Dr. Hobby had not; his study of the cases had been purely clinical. Those who have been so fortunate as to have opportunity to examine those mute from this cause have found lesion of the labyrinth.

---

### SECTION ON LARYNGOLOGY.

### Fourth Day—Morning Session.

The first paper was on

#### NASAL FIBROMATA, by Professor W. E. Casselberry, of Chicago, Illinois. Under this title he included only neoplasms of a purely fibrous or predominating fibrous structure, which originate in the nasal fossæ, anterior to the naso-pharynx, and excluded those of a compound type, prone to occur in this situation, as fibro-myxomata and fibro-sarcomata, and also all those originating in the neighboring sinuses and in the naso-pharynx.

Recent literature has contained numerous reports of cases of naso-pharyngeal fibromata, but fibroid tumors originating primarily, in the nose itself are comparatively rare. The *Internationales Centralblatt für Laryngologie, Rhinologie*, etc., does not contain the report of a single case, and none are included in 265 cases of nasal neoplasm reported by Hopman and Schmiegelow. Mackenzie reported one case of his own, and two others, probably fibromata.

Growths in the nose and naso-pharynx, in a measure correspond in type to the structural elements of the tissue from which they originate. The fibrous layer of the mucosa is especially abundant in the naso-pharynx, hence the frequency of naso-pharyngeal fibromata, while in the anterior nares the superficial mucous layer predominates, and hence the more common occurrence of myxomata and the comparative infrequency in this location of the fibromata. Fibromata may, however, arise from any part of the nasal cavity, as turbinated bodies, septum and floor, but the vault, in the nose proper, is its favorite site.

Local irritation from traumatism or a perversion of the chronic hypertrophic inflammatory process may serve to excite a hyperplasia of the fibrous elements. The early symptoms are of a catarrhal nature, followed by obstruction and distension of the fossa. Nasal bones are forced apart, neighboring cavities encroached upon, eyes protruded and the deformity of "frog-face" possibly produced.

The tumor is not multiple, but may be lobulated, of dark red color, broad base, non-translucent and firm and resistant to the probe.

Microscopically, firm, dense, whitish, and enveloped in a smooth, fibrous capsule.

Microscopically, the fibers are grouped in bundles, or are simply closely interlaced.

Nasal fibromata tend to degenerate into sarcomata and to recur after removal, although if thoroughly extirpated and the base cauterized to the bone the prognosis should be good.

He gave a systematic table of eight cases, including the name of the operator, date, age, symptom, etc., treatment, and result.

In his own case, a lady aged forty years, the left fossa anteriorly was filled by a firm elastic tumor, and there was commencing "frog-face," but no involvement of the autrum or orbit. Septum, anteriorly, was deviated by pressure, far to the right. Naso-pharynx normal. It was removed piece-meal, in weekly sittings, by the galvano-cautery ecraseur, the knife electrode being employed when necessary to prepare a place for the loop to take hold. For the ecraseur he used, instead of platinum, steel piano wire, which on account of its resiliency offers resistance to tissues against which it is pressed, retaining or regaining its contour after contact, thus facilitating envelopment of the part. Under cocaine the operations were painless and comparatively bloodless.

The primary attachments extended along the horizontal plate of the ethmoid bone to the anterior surface of the body of the sphenoid. The patient remainedwell at the end of sixteen months. Of the seven other cases appended, the treatment in case 1, is not stated. In case 2, the tumor projected posteriorly, and was removed by forceps. In cases 3, 4, and 7, disfiguring and somewhat dangerous external operations were performed to give access to the tumor. In case 5, the tumor was successfully removed in fifteen sittings by a cold wire snare. In case 6, attempts at removal by ligature and forceps failed, and an attempt to cut through the base by a bistoury resulted in death from hæmorrhage.

In his own case the galvano-cautery used in the manner described was the one means which rendered a formidable external operation avoidable. But it must not be supposed that all cases will be susceptible of this means of cure. Enormous size and penetration into accessory cavities may render it ineffective.

In such cases electrolysis might serve to reduce the size to such a point that galvano-cautery could cope with it.

Efforts to envelop the whole tumor in the ecraseur for removal at a single operation will usually fail on account of adhesions and tight fit into nasal inequalities.

Mr. Lennox Browne, of London, England, in opening the discussion, said the subject was one of practical interest, and the paper was drawn on the lines on which a paper should be drawn, that is when one listens to such a topic he likes to know at the same time what others have done on the same subject. He could not understand how in modern times one could perform an external operation without first exhausting intranasal means. A similar case he had not observed, but he agreed with the author concerning the treatment.

Professor M. F. Coomes, of Louisville, Kentucky, spoke of two similar cases, but of fibromyxomatous type.

Professor E. F. Ingals, of Chicago, Illinois, said he had not found cocaine in all cases to render operations painless; that the author was fortunate in having so little hæmorrhage, and that he had not been successful in causing the steel wire to heat as readily as platinum.

Professor F. O. Stockton, of Chicago, Illinois, said that a little iridium added to platinum gave the wire the necessary stiffness.

Professor Casselberry, in closing, said individuals differed in susceptibility both to pain and cocaine, still non-success was usually due to solutions too weak or not thoroughly applied. One can use weak solution first to test the patient before resorting to 20 per cent. solutions. The latter should be applied only by cotton applicator, and not by spray, thus avoiding systemic absorption. Perhaps platinum takes the glow from the battery more perfectly than steel, but steel heats for all practical purposes as well as platinum. The steel wire is, however, more liable to burn in two. A fresh piece must be used each time.

AFTERNOON SESSION.

Dr. John O. Roe, of Rochester, New York, then read a paper on

CHOREA LARYNGIS, usually associated with an attack of general chorea. The author gives the history of cases where the affection was purely local as to its manifestation.

Miss S———, aged seventeen, delicate and nervous, after an attack of quinsy, developed a peculiar spasmodic and uncontrollable cough. She would cough rapidly ten or fifteen times, with a peculiar hoarse, barking sound, after which there would be an intermission of from one to three minutes. The cough was in sound so like the barking of a dog that she was the curiosity of the neighborhood, and known as the "barking girl." It did not continue during sleep. He tried electricity and local application without effect. Hypodermics of morphia and atropia produced only temporary relief. Benefit was derived from treating it as chorea. It suddenly ceased ofter seven months.

Another case, a healthy girl of thirteen, with much the same history, was mentioned, where there was no nervousness during the intermissions which lasted one to two minutes.

He mentioned a case of a young woman of sixteen years, with delicate health, where the barking cough continued during meals, and even during sleep, without awakening her. Spray of cocaine increased the trouble. These cases were not associated with uterine trouble. On examining the larynx he found but little congestion or inflammation. Before a paroxysm of coughing there would be a peculiar twitching of the vocal chords for a while, then they would be suddenly approximated and forced apart as the cough occurred. The movements were those of chorea. As a rule, the treatment should be systemic, in addition to local applications—arsenic, valerianate of zinc, iron, valerianate of atropia, hydrocyanic acid, morphia, etc.

Dr. Thorne, of Cincinnati, Ohio, mentioned several cases where chorea laryngis was associated with pregnancy, beginning in second or third month, and ceasing spontaneously just before delivery. He had one case where there was such an alarming spasm of glottis that he thought he would have to perform tracheotomy. The chords were constantly in motion between attacks.

Dr. Lennox Browne, of London, England, has seen a case where the disease appeared with each pregnancy, and disappeared just before delivery. Also three cases where it was cured by the removal of enlarged tonsils. He thinks a sea voyage the very best remedy.

In a paper on

THE DELETERIOUS EFFECTS OF TOBACCO ON THE THROAT AND NOSE,

Professor M. F. Coomes, of Louisville, Kentucky, said, that he considered smoking far more injurious to these parts than chewing. The smoke came into the mouth heated, and loaded with an irritating oil that would soon coat the mucous membrane were it not washed away by the saliva. Cigarette smoking is especially injurious, because the smoke is so universally inhaled, causing pharyngitis, laryngitis, and chronic irritation in the nose, not to mention the injury it may occasion to trachea and lungs. Where the smoke is habitually expelled through the nose, we find hypertrophies, congestion, dilated vessels, and a hæmorrhagic condition. The sense of smell is impaired or destroyed. The potash salts may also have some effect in adding to the injury. Ninety-five per centum of smokers have something abnormal or unhealthy about the upper air-passages. In bad cases, he found chronic hyperæmia and inflammation of epiglottis, with congested chords, and a hacking cough to remove the tough mucus; the voice tires easily.

A peculiar form of tobacco habit is what is known in the South as dipping snuff. It is prevalent among negroes and lower classes of whites. They chew the end of a twig so as to form a rude brush, then they continually dip this into a small box of snuff, and rub it over their gums and teeth. The gums being pushed down on the teeth are red and inflamed, from tobacco lodged between them and the teeth, and the whole pharynx is inflamed.

Professor F. O. Stockton thinks that as a nation we are the greatest chewers, and that chewers suffer most. He does not like potash salts used in the throat or the nose.

Dr. L. Browne smokes cigarettes, and considers them less harmful than any other form, if we don't inhale the smoke, and use a fresh mouthpiece.

The taking of snuff is an especially baneful habit, and likely to cause polypi. Singers should not use tobacco. He excludes potash salts, except, perhaps, the bromide, in treating the upper air-passages.

INTUBATION OR TRACHEOTOMY.

Dr. Max J. Stern, of Philadelphia, Pennsylvania, in a paper on this subject first gave a short history of tracheotomy from the earliest record to the present time. Some of the earlier statistics on the continent of Europe show as high as thirty per centum of recoveries.

In the United States our results have not had such encouraging results, due probably to the difficulty of obtaining consent to an early operation.

He also gave the history of intubation from the first catheterization to the perfection of Dr. O'Dwyer's tubes.

He quoted the following statistics to show the comparative percentage of recoveries: Intubation, 26⅖ per centum; tracheotomy, 26⅖ per centum.

The number of bad results due to accidents was very small in either case. From the following statistics it appears that up to five years of age intubation has decidedly the advantage, over that age tracheotomy has the best record:

| Age. | INTUBATION. Per cent. | TRACHEOTOMY. Per cent. |
|---|---|---|
| Under 2 years | 15 | 3 |
| Between 2 and 2½ years | 24 | 12 |
| " 2½ and 3½ " | 28 7-10 | 17 |
| " 3½ and 4½ " | 33 7-10 | 30 |
| " 4½ and 5½ " | 28 3-10 | 35 |
| Over 5½ years | 37 3-10 | 39½ |

Some of the advantages of intubation are that it is easier to gain parents' consent, does not require skilled assistants or attendants afterward. Less care in the after-treatment is required.

Tracheotomy is extremely difficult in very young children. The usual time that the tube is left in the larynx is four to ten days. As the air can reach the lungs through the proper channels there is less danger to the lungs.

He advises that if the child be under three and a half years, intubation be performed if possible; between three and a half to five, to resort to either; if over five years, perform tracheotomy. Intubation was advised for adults; and that one do not perform tracheotomy if there should be much membrane in trachea. Intubation was recommended for the poor when they cannot get good nursing. Dr. Stern then closed by paying a high tribute to the good work accomplished by the perseverance and energy of Dr. O'Dwyer.

Dr. Charles Denison, of Denver, Colorado, believes in tracheotomy without a tube. He showed a gag which he had modified by removing the long levers that sometimes cause its displacement. It was kept open by a rachet catch.

Professor W. E. Casselberry, in intubation, passes the index-finger not into the larynx, but to the arytenoid cartilages and entrance of æsophagus, instead of trying to lift the epiglottis. He advises wearing a finger-stall and practice on cadaver first.

---

### SECTION ON DERMATOLOGY AND SYPHILOGRAPHY.

### Fourth Day.

Dr. P. G. Unna, of Hamburg, Germany, read a paper, accompanied by microscopic demonstration, on

#### SEBORRHŒAL ECZEMA.

The author does not consider that the diagnosis "acute" or "chronic" eczema is a sufficiently exact and scientific one. For example, there are three distinct types of infantile eczema of the face; viz., a nervous, a tuberculous, and a seborrhœal. The nervous type is seen in the eczema of dentition, appearing upon a perfectly sound skin, usually first upon the cheeks and then upon the forehead. The eruption itches in proportion to the strength of the infant and the thickness of the epidermis. The vesicles, at times, suggest in their appearance and their reddened base herpes zoster, but the marked symmetry of the lesions and the tendency to relapse would prevent such a diagnosis. Seborrhœal eczema is entirely different in that the skin is not in a previously healthy condition. A seborrhœa has existed upon the scalp probably a few weeks after birth, which often spreads over the upper portion of the face after becoming somewhat moist. There is less itching than in the eczema of dentition. For the latter affection a zinc-ichthyol ointment is recommended to be applied and covered with a mask, together with the bromide of potassium, internally, to allay nerve-irritation. In seborrhœal eczema sulphur should be added to the zinc-ointment. Ichthyol is useless, but resorcin acts well.

Dr. Unna regards all so-called dry seborrhœas as chronic inflammatory processes of the skin, and his studies convince him that there is no hypersecretion from the sebaceous glands which can clinically be considered a dry seborrhœa, produced by a deposit upon the surface of the product of these sebaceous glands. The disease which Dr. Unna has named eczema seborrhoicum is dependent upon a condition of, or rather change in, the coiled or sweat-glands, giving rise to the secretion of fat by them. An increase of fat upon the surface indicates increased activity when it proceeds from the sebaceous glands, but when the sweat-glands pour out fat it is because of the death of their endothelial cells.

Almost all seborrhœal eczemas have their starting point in the scalp. Three forms are described. In one, eczema seborrhoicum begins as a latent catarrh of the scalp, and goes through the stages of scaly formation and dryness, finally ending as a hyperidrosis oleosa. In the second, the scales heap themselves up between the hairs into fatty crusts, and cause the hairs to fall out. A corona seborrhoica upon the forehead, at the margin of the hair, is described, which is typical of this form of the affection.

The third form is that in which the catarrhal appearances are the most pronounced and in which "weeping" occurs, especially about the temples and eyes. This follows a simple itching pityriasis, and produces the appearance of a moist, shining eczema. A pityriasis or a seborrhœa may exist upon the scalp, while at the same time an eczema is present upon the face. The chest is affected with the crusty form almost exclusively.

Upon the arms there is a predilection for the flexor surfaces, which is explained by the rôle the sweat glands play in eczema seborrhoicum.

Upon the legs, early in the disease, we find only the large papular and crusting forms.

Patches of seborrhœa about the mouth and nose of old people are often the starting-points of carcinoma. In almost all cases of eczema seborrhoicum of the scalp, a simple, itchy scaliness of the ear is observed. A patch of eczema seborrhoicum is extremely stationary, remaining for years possibly without much change in size and giving rise to only slight symptoms. Beginning upon the scalp or head, as a rule, it extends to other parts below in a very gradual manner; especially locating upon the face, sternal, and intra-scapular regions. This course is so often the rule as to be regarded as pathognomonic, as no other eczema or psoriasis runs this course.

When the whole body becomes involved it resembles closely pityriasis rubra. Psoriasis is often confounded with seborrhœal eczema. The latter attacks the parts near the median line of the body, and is more stationary than psoriasis. It is preceded by a local seborrhœa, and the scales and crusts have a decidedly fatty character. The patches have also a peculiar configuration and spontaneously flatten out in the middle or upon one side. The prognosis is more favorable than in psoriasis. The cure is not easy, because the deep-seated sweat-glands are implicated. Sulphur is the great remedy for the disease. More active remedies for the scaly and crusty forms are pyrogallol and resorcin. Internal treatment is seldom required. Once cured, prophylactic measures, such as hygiene of the skin must be employed.

In the discussion which followed, Dr. J. Zeisler, of Chicago, Illinois, said he was impressed with the closeness of Dr. Unna's observations. Such cases are frequently seen here, and are called seborrhœa. He had regarded it as a hypertrophy of the endothelium of the sebaceous glands.

Professor G. H. Rohe, of Baltimore, Maryland, said no one was in a position to call in question any statements or claims made by Dr. Unna, as similar work had not been done by others.

Professor Robinson, the president, said there is much difficulty in classing eczema on account of its great varieties, causes and modifications. As regards seborrhœal eczema, seborrhœa and eczema existing together is not uncommonly observed. He can understand how they can go hand in hand, but does not see how the fat can have an effect as a local irritant in producing an eczema.

In closing, Dr. Unna said the point had been misunderstood; that he did not believe the fat was the cause of the irritation. He had merely spoken of the clinical side of the question. He was at the present time experimenting with some thirty cultures of micro-organisms from seborrhœa, and believed that a parasite was the cause of the irritation at a point where the fatty matter existed.

What we have called seborrhœa sicca is not a hyperscretion, but an inflammatory affection.

Warts of the scalp and of the penis he also thinks are due to a parasite which finds its pabulum upon the skin of the penis and also upon the fatty matter from a seborrhœa of the scalp.

MULTIPLE SARCOMA OF THE SKIN.

Professor G. H. Rohe, of Baltimore, Maryland, presented a patient with this rare disease, characterized by the development within the skin of multiple sarcomatous tumors. The patient was a man of about thirty years of age. The tumors were of about the size of an English walnut, and were situated upon the regions of the back and shoulders, legs and thighs. He was also suffering from tuberculosis of the lungs, and had also partial hemiplegia.

Professor A. R. Robinson, the president, gave a report of

A UNIQUE CASE OF PROGRESSIVE MELANOSIS OF THE SKIN, the peculiarity consisting in the progressive nature of the affection, its long duration, and the situation of the pigment. The patient was a female who, twenty-one years ago, at the age of eight, first noticed a small, dark spot upon the side of the temple. There were no subjective symptoms of pain or tingling in the part. She had suffered from chills and fever. The lesion consisted of pin-point-sized non-elevated dark or bluish-dark spots, which formed a patch occupying the whole lateral surface of the forehead.

Microscopical examination of the skin showed the epithelium to be of normal thickness. The rete mucosum contained dark-brown pigment-granules, which were also seen in lower rows of the pavement epithelium.

Discussing the paper, Dr. Unna said he had been impressed with the blue color of the patch as represented in the colored drawing contrasting with the dark-brown color of the pigment seen under the microscope. He asked if clinically the disease presented this blue color, and whether the corneous layer was especially thick. He had never seen a similar case, and regarded it as interesting and unique.

Professor Robinson answered that the color was faithfully represented and that the corneous layer was not thick.

A paper was then read by Dr. A. H. Ohmann-Dumesnil, of St. Louis, Missouri, on

DOUBLE COMEDO.

Dr. Dumesnil said that he had found double comedo in two and one-fourth per cent. of all male patients in hospital practice. It occurs upon all portions of the body excepting the limbs and forehead.

In regard to the formation, he does not regard it as congenital, but that two contiguous comedones become fused by the absorption of the intervening septum. Multiple plugs of sebaceous matter are found, having only one central common cavity and possibly three or four external openings, all connected within. This is readily demonstrated by cutting the bridge of skin between them.

The mode of production is the same as in the single comedo, and the cavity is the analogue of the single form. This explanation he regarded as more reasonable than to suppose the existence of a number of anomalies in comedo-formation, with the necessity of having an anomaly to account for each new case.

Dr. Unna, in discussing the paper, said he believed that double comedo never developed in a totally healthy skin—that at some time there had been an inflammatory process followed by cicatrization. He had observed many cases and always found evidences of these changes, and a scar could usually be discovered.

Dr. Dumesnil did not believe in the inflammatory process theory, and had not observed scars in many cases. He hoped some one would be able to make observations upon the disease in the formation-stage.

The next paper was by Dr. H. Watraszewski, of Warsaw, Poland, on

TREATMENT OF SYPHILIS BY INJECTION OF INSOLUBLE MERCURIC SALTS.

There is nothing new in the injection of insoluble salts of mercury in syphilis. The author has already made a communication on the same subject. He has since pursued his investigations and has been led to give the preference to the yellow oxide of mercury. The injections are to be made about once each week, and from four to five injections have been found sufficient to cause a disappearance of the symptoms. He has never had any difficulty from abscesses.

Cases come to the special hospital for syphilis, with which he is connected, from all parts of Poland, having been treated by other methods. Some have the disease in its worst forms, and present all the conditions favorable for a thorough test of this treatment. The author has had results follow the injection-cure which are very favorable and warrant his enthusiasm. The feature of the method is the small number of injections required, twelve to twenty being required for a course of treatment, and only four or five to cause a disappearance of the lesions at any time present.

Once each week a Pravaz syringeful of the following solution is to be injected deeply into the tissues.

℞. Hydrargyri oxyd. flav.........1.0 gram.
Gummi arab. ............0.25 centigram.
Aquæ destillat...........30.0 grams.

M. S.—Shake and inject.

A Pravaz syringeful represents about four centigrams, or two-thirds of a grain.

A calomel-solution made in the same way, but three times as strong, has been used by the author, who finds that while it is beneficial the reaction and irritation caused by it are much greater.

In the discussion, Dr. Gottheil, of New York, N. Y., said that during the past few years we have had brought forward a variety of new methods for the injection of the mercuric salts. He thinks that patients in other countries must differ from those in America, for here they will not submit to this method. Internal treatment, he believes, must here at least remain the more common course. He asked if the injection-course was employed only in the severer cases or as routine treatment.

Professor J. V. Shoemaker, of Philadelphia, Pennsylvania, said he had treated many cases of syphilis by this method during the past seven or eight years, and held it in high esteem, especially for old, chronic cases, where treatment had failed. He made the injections in the arm, or wherever there was plenty of fatty tissue, in the inferior scapular region and the back, and deep into the muscular tissue of the gluteal region when the patient was thin. He employed the bichloride in a watery solution, and gave as high as half-grain and even grain-doses when the patient had been under treatment for some time, making the injections once in about three days. He had never seen an abcess result.

Dr. Klotz, of New York, N. Y., said he had had considerable experience with the hypodermic method, both in public and private practice, and he was surprised to hear the remarks of Dr. Gottheil relating to an inability to employ it with patients in this country. His private patients had never objected, when they were intelligent men and the process had been explained to them. In fact, when a relapse occurred they would voluntarily return and ask for it. There is some pain, but the patient will stand it, and the results compensate for it.

Dr. J. Zeisler, of Chicago, Illinois, was surprised to hear what Professor Shoemaker had said. He had considered that the salts should be injected daily. The more we give at a dose the greater is the danger, and he asked if such a large dose as a grain was not very dangerous to the life of the patient. In Vienna, when he had used the method, the rule was to give one centigram in the syringeful. He thought the good feature of the method proposed by the reader of the paper was the small number of injections necessary to get the good results claimed.

Professor Shoemaker, in answer, said that some one in London had reported fifteen hundred cases treated, in which one-third grain of bichloride had been the dose used.

He usually began with one-tenth grain, and increased it until the physiological action had been produced, or the larger doses used.

Dr. Watraszewski, in closing, said that we must not use the hypodermic method indiscriminately, but must individualize. He saw each year many of the worst imaginable cases. The patient would throw pills out of the window instead of taking them. Inuctions would be improperly and imperfectly performed; but here we have an accurate, sure, and safe means of dealing with all cases, and in severe cases the cures were much more rapid than by other means. It has proven especially beneficial in his hands in syphilis maligna of finger, and in late syphilis.

---

### SECTION ON PUBLIC AND INTERNATIONAL HYGIENE.

### Fourth Day.

A paper by Tomassi Crudelli, of Rome, Italy, on

FACTS AND THEORIES RELATING TO THE CAUSE, NATURE AND PREVENTION OF MALARIAL FEVER, was read and the bacillus described.

Dr. George T. Maxwell, of Ocala, Florida, then read a paper on

THE INFLUENCE OF CLIMATE ON THE PRODUCTION OF CHOLERA INFANTUM, in which he took the position that heat was an essential factor in its causation. The general scope of the paper was that the cause of this disease is not yet well understood.

THE HISTORY AND PRACTICAL APPLICATION OF STEAM AS A DISINFECTANT, was the title of a paper read by Dr. A. N. Bell, of New York, N. Y., which was followed by papers on "The Sanitary Inspection of Railroad and Passenger Cars," by Dr. R. Harvey Reed, of Mansfield, Ohio; "Public Hygiene," by Dr. W. C. Cook, of Nashville, Tennessee; "The Clinical History of Continued Malarial Fever," by Dr. B. D. Taylor, U. S. Army; "A New Method of Detecting Trichina Spiralis in Meat," by James A. Close, M. B. (Toronto), L. R. C. S. E.

### SECTION ON CLIMATOLOGY AND DEMOGRAPHY.

### Fourth Day.

Dr. Titus Munson Coan, of New York, N. Y., read a paper on

AMERICAN MINERAL WATERS, WITH REMARKS ON CLIMATE.

He discussed the subject under four topographical headings: Springs of the Atlantic, the Southern Central, the Northern Central, and the Western or Pacific States. He called attention to the fact that much of the Eastern area of the country was of earlier formation than that of Europe; that, in fact, this part of the New World was, geologically speaking, the Old World, and not the New. This fact explained the comparative absence of thermal springs in the East, while the Western area, including but thirty-nine per centum of the total area of the country, yet contained eighty per centum of the known thermal springs. The range of the American mineral springs in their chemical constitution is very great, and their curative waters are as important as those of Europe. In some parts of the country, as in New Mexico and in Nevada, it is often easier to find an alkaline or a saline spring than a stream of pure water. Dr. Coan gave a rapid survey of the subject, mentioning the typical springs which are suitable for the cure of particular diseases, and at which good hotel or other accommodation can be found. He remarked upon the comparative absence of well-appointed *sanatoria*, or cures, at the American springs, and the consequent lack of systematic treatment, leading both patients and physicians to underrate the curative value of spring-treatment. Climate is an adjunct element of the utmost importance in spring-treatment; and the best climate in the country is that of California and Oregon. The climate of the Hawaiian Islands is probably the most equable that is known, at the comfortable range of about 70° to 80° F., and those islands are destined to become a health resort for Americans.

Dr. Richard J. Nunn, of Savannah, Georgia, read a paper entitled

A CONTRIBUTION TO THE STUDY OF CLIMATIC AND OTHER PECULIARITIES OF LOCALITIES WHICH DETERMINE EXEMPTION FROM ENDEMIC PLAGUES.

The object of Dr. Nunn's paper was to determine whether the extraordinary exemptions from certain diseases in Savannah are due to any special topographical or sanitary conditions in that city.

Vesical calculus, typhoid fever, typhus fever, puerperal fever, are absent; Asiatic cholera visited the city only once (1866); diphtheria and exanthematous diseases are mild; membranous croup is exceedingly rare; cholera infantum is mild and exceedingly rare; cerebro-spinal meningitis has appeared but once in the city; sunstrokes are very unusual; erysipelas is not common; yellow fever has occurred but four times as an epidemic; dengue is infrequent.

The varying death-rate between the white and black races was referred to. Between 1856 and 1860 the black mortality never reached that of the whites, while since the war the death-rate among the negroes has reached double that of the whites. The changed social condition of the black race is supposed to be responsible for this variation. Formerly the negroes were almost entirely exempt from consumption, now they are extremely liable to this disease. The same may be said of syphilis.

The following papers were then read:

"The Injurious Effects of Overcrowding in Cities," by Dr. A. Wernich, of Coslin, Germany; "The Thermometer as a Climatological Instrument," by Major Charles Smart, Surgeon United States Army; "Vital Statistics and Medical Geography," by Alfred Haviland, M.R.C.S., of London, England; "Western North Carolina as a Health Resort," by Dr. Henry O. Marcy, of Boston, Massachusetts; "Therapeutic Influences of the Climate of Southern California," by Dr. P. C. Remondino, of San Diego, California, and "Short Notes on the Mineral and Thermal Springs of California," by Professor W. F. McNutt, of San Francisco, California.

---

### SECTION ON PSYCHOLOGICAL MEDICINE AND NERVOUS DISEASES.

### Fourth Day.

Dr. S. S. Bishop, of Chicago, Illinois, read a paper on the

#### PATHOLOGY OF HAY FEVER.

He believed that full reliance could never be placed in the pollen theory. The very fact that hay fever recurred upon the very same day of the year in many cases, irrespective of the late or early coming of the seasons, was a proof against it. He believed it was of nervous origin, and would designate it by the term nervous catarrh.

Dr. Channing thought it should hardly be dignified with the term of neurosis. He supported the pollen theory, and stated that removal to mountains or sea-shore resorts was an effectual remedy in the cases he had seen.

Dr. Brush, of Philadelphia, Pennsylvania, did not believe in the pollen theory. He had himself suffered from hay asthma at different times in the summer, from July until autumn.

Dr. Hurd believed in the neurotic origin of hay fever until the present season, which he said had been an unusually hard season for sufferers from nervous troubles. As a matter of fact, hay-fever invalids had had little or no discomfort. He believed ragweed to have a potent effect in causing hay fever, and stated that in Michigan this year ragweed had not flourished, and the result is seen in the little discomfort from hay asthma.

Dr. Andrews, the president, believed we could point to no one cause for hay fever. Different causes effected different people. He would ask Dr. Bishop for his views on the treatment of hay fever.

Dr. Bishop stated that people of a particularly nervous temperament were the greatest sufferers from hay fever, just as they were often great sufferers from peculiar troubles of nervous origin. He had heard a patient declare that eating strawberries would throw her into convulsions. Hay fever is most prevalent, he said, during the most depressing season of the year. He could not believe in the pollen theory.

As to the treatment, he would say that it was more palliative than curative in the majority of cases. One physician of his acquaintance had procured benefit in forty-five cases by the application of the galvano-cautery to the septum nasi. He had obtained either partial or complete relief.

As to his own practice, he had found the use of a combination of sulphate of morphia and atropia, in the proportion of $\frac{1}{150}$th of a grain of atropia to one-half grain of sulphate of morphia, to have a most beneficial effect. Not that he would give one-half grain of morphia to patients at once. He had made this combination of morphia and atrophia into small tablets, and gave them in the proportion of one-eighth of a grain of morphia to a proportionate amount of atropia.

In his experience, if a patient would take one or two of these tablets immediately upon feeling the first symptoms of hay fever, it would usually abort the attack. He had always given as large a dose of morphia as he thought the system of the patient could stand, in order to make an impression at once upon it. He had also found taking cool drinks or ice-cream immediately before going to bed to be of great assistance in warding off nocturnal attacks.

If a patient keep himself cool throughout the day, too, there is less danger of his being attacked. Quinine deadens sensibility, and is of use, but will not always avert an attack.

The next paper was by Dr. Elliott, of New Haven, Connecticut, on

THE TREATMENT OF NEURALGIA, by the general practitioner.

Dr. Crego did not like to hear that Dr. Elliott prescribed so much morphia in the treatment of neuralgia. It is the duty of physicians to use as little morphia as possible. Arsenic and iron should be prescribed, and electricity be used as often as practicable.

Dr. Duquet thought well of the hypodermic injections of chloroform in sciatica. He had found injections of twenty drops very deeply into the muscles, just at the beginning of an attack, of great value. He did not share in Dr. Crego's sweeping denunciation of morphia.

Dr. Heber Ellis thought highly of hydrochlorate of ammonia, which he said had been used with success in Germany, and which he had himself found valuable. He could not so highly recommend quinine. He had seen cases of chronic deafness result from its constant use.

Dr. Gristrom, of Sweden, had found massage of great value in the treatment of neuralgia.

Professor D. R. Brower, of Chicago, Illinois, discouraged the indiscriminate use of morphia and quinine. He had seen too many morphia *habitues*, whose habit had commenced in this way, not to feel like protesting against its too frequent use. One may obtain a great amount of *èclat* for instantly relieving the patient, but do him, in many cases, far more harm than good.

Dr. Clark, of Toronto, Canada, said he took good care when prescribing morphia, in all cases, to conceal from the patient the fact that he was giving him morphia. Many times when prescribing it internally he gave it with some medicines of such nauseating bitterness that the patient was rarely inclined to ask for its like again. He believed that the treatment of neuralgia should depend upon the causes of the disease.

Dr. Andrews, the president, recommended the use of cod-liver oil in the treatment of chronic neuralgia. In an emulsion by adding phosphites, iron, arsenic, and other tonics, it was excellent. The use of the hot water bag, too, was very useful.

Dr. Russell read a paper entitled

BORDERLAND OF INSANITY, endeavoring to show that epilepsy and kindred diseases did not necessarily produce mental weakness. He instanced Julius Cæsar, Napoleon, Martin Luther, Cowper, and others.

Dr. Gundry said we had no exact facts of any kind as to the nervous troubles from which Cæsar, Napoleon, and others, were said to suffer.

Dr. Porter hoped the suggestions of Dr. Russell's paper would be followed. In too many cases were the obvious signs of insanity not heeded, and oftentimes a patient is permitted to remain at home, passing without treatment into a case of chronic insanity.

Dr. Ellis, of London, England, thought that the course pursued in the past, both in England and America, in the commitment of patients to asylums, was not such as to induce the friends of a patient to place him in an asylum unless in many cases absolutely compelled to do so. Happily, in England a new plan had been adopted. A medical man receives one or two patients into his house and these patients are seen regularly by the commissioners and receive proper inspection. They secure the benefit of change of air, of environment, of diet, and are at all times under careful personal observation by the physician.

When a patient is cured he retires quietly to his home, and does not retain with him the unpleasant recollection that he has been committed to an asylum—a humiliating thought under the best of circumstances. The great objection to this plan was the expense involved to the patient.

Dr. Fisher read the next paper, written by Dr. Edward Cowles, entitled

NURSING REFORM, which detailed the success which he has had at the McLean Asylum in training his nurses, and of the improved *esprit de corps* among the attendants and patients.

AFTERNOON SESSION.

Dr. W. W. Godding presented a paper entitled

INSANITY AS A DEFENSE FOR CRIME.

He denied that the test of a knowledge of right and wrong was a correct one to apply in determining the criminal responsibility of lunatics. Judges in the United States have made but a metaphysical study of insanity. They should obtain clinical knowledge of cases by visiting a hospital for the insane, and thus get rid of the idea that lunatics act reasonably in regard to their acts.

He believes that the New Hampshire ruling as to insanity, which Judge Cox on the Guiteau trial rejected as heresy—namely, that all questions on insanity were facts for the jury to determine, and not as rulings for the court—would one day prevail.

Dr. Savage then opened the discussion upon

SYPHILIS ASSOCIATED WITH GENERAL PARALYSIS.

General paralysis is not a definite disease, but a degeneration. Syphilis seems to have a distinct and special tendency to cause degeneration of the nervous structure. There is a difference in statistics as to the numerical relations between syphilis and general paralysis. He believed that very many general paralytics had had syphilis, and that more general paralysis was due to syphilis.

He could not distinguish between general paralysis due to inherited weakness and that due to syphilis and that from any other cause. General paralysis associated with syphilis might begin in the brain or cord. Acute general paralysis might suddenly develop in cases of chronic syphilis. Dr. Savage gave a very interesting case of this form, in a man in whom syphilis had not manifested itself for several years, and in whom general paralysis had suddenly developed and gone on to a fatal termination in very brief time.

General paralysis with syphilis may run an ordinary course in every way, having no special cranial-nerve or other complication. In some of these cases the patients have a wan aspect, with capillary congestion about the face. There are very many cases in which the following history is given—viz., that there has been a cranial-nerve lesion which has improved under specific treatment, and later, symptoms of progressive degeneration have set in.

The nerve-lesion may be motor or sensory. He had had cases of ptosis followed by general paralysis, and of giddiness followed by general paralysis.

In cases of general paralysis associated with syphilis, some are spinal, and of these some are ataxic; others have degeneration in the lateral columns, with spastic symptoms. There may be eye troubles with either. The course which cases of general paralysis with syphilitic histories run differs in some particulars from others; it appears to be more subject to remission, and even to apparent cure.

The Section next listened to an address by Professor Mendel on

MORAL INSANITY.

He maintained that the term should be stricken from the nomenclature of mental diseases. Mental imbecility or paranoia, or something more tangible, should be adopted.

Dr. Savage said there were certain terms which we were at times obliged to use provisionally, and this was one of them. There were people with moral scars who did not deserve the term paranoia nor imbecile, unless we extend the term unduly. Of course I agree with Professor Mendel that we must be careful not to use in courts of law terms that we cannot define, but I still believe we shall have to use the term moral insanity.

Dr. Hughes did not question the existence of moral insanity, as described by Pritchard. He would substitute for the term moral insanity, because of the difficulty encountered in explaining its meaning in courts of law—"a state of imbecility associated with congenital mental defect."

The discussion upon the relation of syphilis to general paralysis was then resumed.

Professor Mendel's views were presented in German.

Dr. Mickle had often seen cases due to syphilis present the same symptoms as the ordinary every-day case of general paralysis. Other groups were those presenting at first local motor symptoms, local motor paralysis, or sensory symptoms, as well as nocturnal headache. There are local unilateral spasms, followed by paralysis. These paralyses clear up sometimes, but their more usual course is to gradually take on a complete hemiplegia. Such patients often present local symptoms late in the course of affection, such as ocular paralysis. Then when one comes to the necropsy, there is in this class of cases a pachymeningitis—a local one—affecting the dura on one side. Thickening if found on one side, and there is adherence of the meninges to the surface of the convolutions and erosion of the convolutions, which is so common in general paralysis.

There is often a somewhat diffuse yet circumscribed sclerosis affecting a greater or less tract of the cortex of the brain. That I have frequently found existing upon one side chiefly, or upon one side only. There is another group of cases of brain-syphilis and general paralysis. The affection often simulates its demented form. The patient has probably epileptiform convulsions, a local paralysis, more or less marked, and sometimes a monoplegia. Such patients often die in epileptiform seizures, and in an epileptic status. At the necropsy one finds the cerebral blood-vessels, particularly those of the circle of Willis, with their coats enormously thickened. Then they exhibit sometimes growths that are really syphilomatous, really a gumma of the arterial coat. At such times there is a diminution of the lumen of the vessels. This explains the degeneration which ensues in these cases.

In consequence of syphilitic arteritis, affecting the walls of the large blood-vessels, and in consequence of the syphiloma affecting their coats, and the thickening of the walls, there is an obstruction of the walls and a tendency to thrombosis, and as a result we get local softenings, and these give symptoms of ordinary paralysis. In these cases there are gummatuous infiltrations affecting other than arterial walls.

Dr. Down said that in his experience in London hospitals he had come to the conclusion that nearly all cases of locomotor ataxy were of syphilitic character, that they all responded to specific treatment, and that they are, as a rule, syphilitic. He had had an opportunity of studying the question whether there was any connection between locomotor ataxy and general paralysis of the insane. He cited the case of a chaplain in a Welsh prison who had come under his observation suffering from locomotor ataxy of a progressive character. There was nothing else indicating trouble, but this man, after ten years, had some exaltation of mind which rapidly increased, and he soon became a general paralytic and died in about eighteen months after the first attack.

Dr. Yellowlees said one often sees cases of general paralysis in which there was constitutional syphilis running an ordinary course. He thought one should be careful as to stating the effect of syphilis in the history of the disease. He agreed emphatically with what Drs. Savage and Down had said about the probable syphilitic origin of those cases which begin with spinal symptoms, but we seem to have as a result in cases of general paralysis occurring in patients with a history of constitutional syphilis that the disease is modified to a greater or less extent by a greater tendency to local paralysis than in ordinary cases.

Dr. Nichols said in the large number of cases of general paralysis which he had treated some two-thirds, he thought, had had syphilis. He had really doubted whether syphilis was an essential cause of general paralysis of the insane. It seemed to him that those cases which he had not been able to trace to a syphilitic origin ran their course more regularly than those in which he could, and he had never been able to benefit a patient who had not had syphilis by any anti-syphilitic treatment, and he had retarded the progress of the case in many instances in which he knew that the patient had had syphilis. It had appeared to him that in some way the degeneration of the brain did take on a syphilitic character, although it was accompanied undoubtedly by gross lesions that were common to other forms of brain-degeneration. He might add that he had supposed that excessive venery, excessive intellectual labor, and loss of sleep, were the most efficient causes of general paralysis of the insane, and it seemed to him that they would cause it independently of syphilis.

Dr. Spitzka was obliged to differ very materially from the conclusions of the distinguished superintendent of the Bethlehem Asylum in some particulars. It was a new suggestion to him that all cases of general paralysis of the insane where spinal troubles, such as locomotor ataxy, precede the mental disturbance are syphilitic in their origin. One constantly growing evil seen in all large capitals was the habit of imperfect coitus indulged in for the prevention of conception. This has a most deleterious effect upon the spinal apparatus. Another was the vicious habit indulged in by those who are losing sexual power. These certainly were causes of locomotor ataxy. Dr. Spitzka thought that, notwithstanding the objections made yesterday and to-day, that we were justified in adopting the term syphilitic dementia. We are shown, in insane hospitals, cases of alcoholic dementia and cases of puerperal insanity.

Now, can you tell the characteristic symptoms of puerperal insanity ? It is known in the peculiar combination of symptoms as a whole. Syphilitic dementia differs from ordinary paralytic dementia, or paretic dementia. He had seen hæmorrhages in brains, but not from those places in which they were usually found in cases of status epilepticus. Wendel in his monograph has pointed out many of the peculiarities by which we are able to differentiate cases of syphilitic dementia from ordinary cases of general paralysis of the insane, that there are changes found in ordinary cases of general paralysis that are not seen in cases of syphilitic dementia Dr. Spitzka then enumerated several differences which he found in post-mortems between these two classes of cases. In the matter of nomenclature, he said, we often strain at a gnat and swallow a camel. He could not see why this distinctive term should not be adopted. He believed wine, women, and worry the most potent factors in causing general paresis.

Dr. Hughes asked if any one present had made post-mortem examination of general paralytics who had died during remissions of the disease, particularly in cases where the patient had suffered from his first attack of insanity, and had died during the first remission ?

Dr. Brush said he had seen just such a case—a patient who had died of phthisis during the first remission of the disease. The leisons usually found in general paralysis of the insane were found in this case. Dr. Brush was not sure but that the phthisis had acted in this case in bringing about the remission in much the same way as in the case mentioned by Dr. Savage, where the carbuncle had developed between his patient's shoulders.

Dr. Hurd believed we had all seen cases of general paralysis of unmistakable syphilitic origin. He would ask Dr. Savage, if he believed any benefit would ensue in these cases from anti-syphilitic treatment?

Dr. Savage in reply cited the case of a patient who had suffered from paresis of a syphilitic origin, and who, after being put upon anti-syphilitic treatment, enjoyed a remission after a fortnight. He believed this a fair specimen of the results to be found in these cases. Certainly if these cases are not cured it seems to be the consensus of opinion that they are relieved. He believed that we have reached the high tide of syphilitic pathology, and that it was now time to mark a shore line. Dr. Savage did not like to have too definite lines drawn in the nomenclature of paralysis. Since we knew comparatively little of the relationship between syphilitic dementia and syphilitic general paralysis, we had better not be too definite.

---

### SECTION ON DENTAL AND ORAL SURGERY.

### Fourth Day—Morning Session.

#### MICROSCOPY.

Professor Frank Abbott, of New York, N. Y., and Dr. R. R. Andrews, of Cambridge, Massachusetts, were in charge of this department. Every facility was afforded the members of this section to acquaint themselves with dental microscopy, both physiological and pathological. Among the ground specimens shown by Professor Abbott were those of carious teeth, congenital pathological enamel, hyperostosis (osteomas) of the roots of teeth, and deposits of secondary dentine. Dr. Andrews exhibited serial slides of the developing teeth, and the development of the dental fibril. About forty negatives from his photomicrograph were especially interesting and valuable.

#### CLINICS.

About thirty gentlemen gave clinics in filling teeth with gold, pivoting teeth, constructing artificial dentures, and treating (surgically) diseased conditions of the gums.

Dr. C. L. Goddard, of San Francisco, California, read a paper entitled

#### PAIN IN THE TEMPORO-MAXILLARY JOINT CAUSED BY IRREGULARITY OF THE TEETH.

A patient, thirty years old, experienced pain in temporo-maxillary joint during mastication, which was caused by straining the muscles and ligaments, owing to masticating with the jaw protruded. When the teeth were brought together, as in the act of masticating the incisors alone touched, and the bicuspids and molars were about one-eight of an inch apart. The treatment employed consisted in spreading the upper teeth and thereby securing a proper articulation. Dr. E. A. Chisholm, of Tuscaloosa, Alabama, read a paper entitled

#### THE INFLUENCE OF WEATHER CHANGES ON THE HUMAN ORGANISM.

After carefully noting the influence exerted by temperature, humidity, and electricity, the author concludes that by far the greatest power over human organism is exerted by atmospheric pressure. In support of this theory he submits two arguments. The normal atmospheric weight on man is 14.7 pounds to the square inch at the sea level. The body is sustained by an equal power of resistance, wisely provided. If the pressure be less, the surface of the body will be distended, and the superficial circulation less restrained. This change can be brought about by exposure to great altitude, as well as by natural physical causes, when the circulation will be disturbed just the same. Any undue pressure on a portion of the body may then be felt. May not this disturbance of tension on soft tissues which are fixed to the bony framework of man, or where disease has a seat in periosteal and ligamentous attachments, be liable to greater inflammation? Or when the pulp of a tooth, which in a state of health is inclosed in a bony chamber (which has no expansive liberties, nor needs them as long as health continues), becomes exposed through a small aperture; when the normal atmospheric balance is lowered, the nerve has a tendency to be drawn through the aperture and takes on inflammation, probably followed by congestion and complete devitalization.

A report from the Pennsylvania Hospital, some years ago, on the observation of barometric pressure in surgical operations, shows that in 259 operations the barometer was ascending in 102, descending in 123, and standing in 34. Fifty-four of the whole number were fatal, 11 having been operated on with barometer ascending, 25 when descending, and 8 when standing.

---

### Afternoon Session.

The Section was honored by a visit from the President of the Congress, Professor N. S. Davis. In introducing him President J. Taft recounted the efforts that had been put forth by Professor Davis to secure recognition to the Dental Section. To him more than to any other man in the medical profession is due the credit for having removed the obstructions in the way of the dental specialty.

Professor Davis replied: Twenty-two years ago I had the pleasure of entertaining the members of the American Dental Association, then assembled at Chicago. On that occasion I expressed the hope that some day, in the near future, we might meet on equal grounds. My hopes of that day are realized to-day. At the last meeting of the American Medical Association, when the question was brought up to admit dentists holding their degree from a recognized institution, it met with no opposition. The action of that body has forever removed the obstacle which had been in your way, and you are now on an equal footing with your medical brethren. He congratulated the members for the interest they took in the advancement of the healing art, and closed by warning them not to fall into "schools," but to meet everyone on the broad field of science.

Professor E. S. Talbot, of Chicago, Illinois, read a paper on

ETIOLOGY OF IRREGULARITIES OF THE JAWS AND TEETH.

This paper was very exhaustive and thoroughly well prepared. The writer showed throughout an intimate acquaintance with the subject, and the Section was not lacking in appreciation of his efforts.

The paper was discussed by Dr. W. C. Barrett, of Buffalo, New York.

A paper by Dr. J. J. R. Patrick, Belleville, Illinois, on "Irregularities," was read by title; also one by Dr. E. H. Angle, Minneapolis, Minnesota, entitled "Notes on Othodontia, with a New System of Regulation and Retention;" also by Professor L. C. Ingersoll, Keokuk, Iowa, on "Inflammation of the Oral Tissues."

*To be Concluded in November Number.*

